# Long noncoding RNA PiHL regulates p53 protein stability through GRWD1/RPL11/MDM2 axis in colorectal cancer

**DOI:** 10.7150/thno.36045

**Published:** 2020-01-01

**Authors:** Xuan Deng, Sihan Li, Fanyang Kong, Haoyu Ruan, Xiao Xu, Xinju Zhang, Zhiyuan Wu, Lin Zhang, Ying Xu, Hong Yuan, Haixia Peng, Da Yang, Ming Guan

**Affiliations:** 1 Department of Laboratory Medicine, Huashan Hospital, Shanghai Medical College, Fudan University, Shanghai 200040, China;; 2 Center for Pharmacogenetics, Department of Pharmaceutical Sciences, University of Pittsburgh, Pittsburgh, PA 15261, USA;; 3 Department of Gastroenterology, Changhai Hospital, Second Military Medical University, Shanghai 222300, China;; 4 UPMC Hillman Cancer Center, Pittsburgh, PA, 15213, USA;; 5 Digestive Endoscopy Center, Tongren Hospital, Shanghai Jiaotong University School of Medicine, Shanghai 200050, China;; 6 Department of Clinical Laboratory, The First Affiliated Hospital of Dalian Medical University, Dalian 116011, China.

**Keywords:** Long noncoding RNA, Colorectal cancer, p53

## Abstract

We identified a novel long noncoding RNA (lncRNA) upregulated in colorectal cancer (CRC). We elucidated its role and clinical significance in CRC carcinogenesis.

**Methods:** LncRNA candidates were identified using TCGA database. LncRNA expression profiles were studied by qRT-PCR and microarray in paired tumor and normal tissues. The independence of the signature in survival prediction was evaluated by multivariable Cox regression analysis. The mechanisms of lncRNA function and regulation in CRC were examined using molecular biological methods.

**Results:** We identified a novel long noncoding gene (PiHL, P53 inHibiting LncRNA) from 8q24.21 as a p53 negative regulator. PiHL is drastically upregulated in CRC and is an independent predictor of CRC poor prognosis. Further *in vitro* and *in vivo* models demonstrated that PiHL was crucial in maintaining cell proliferation and inducing 5-FU chemoresistance through a p53-dependent manner. Mechanistically, PiHL acts to promote p53 ubiquitination by sequestering RPL11 from MDM2, through enhancing GRWD1 and RPL11 complex formation. We further show that p53 can directly bind to PiHL promoter and regulating its expression.

**Conclusion:** Our study illustrates how cancer cells hijack the PiHL-p53 axis to promote CRC progression and chemoresistance. PiHL plays an oncogenic role in CRC carcinogenesis and is an independent prognostic factor as well as a potential therapeutic target for CRC patients.

## Introduction

To facilitate aberrant proliferation and cell survival during tumor progression, a number of genetic alterations are typically selected for in cancerous cells 1. Among these alterations, somatic copy number variants (CNVs) play important roles in various cancers, including CRC 2, 3. Although mammalian genomes are widely transcribed, the vast majority of these transcripts are non-coding RNAs (ncRNAs), among which are long non-coding RNAs (lncRNAs) with a length of over 200 nucleotides 4. Studies have pointed to the emerging roles of lncRNAs locating at these aberrant chromosome regions in tumor development. For example, the copy number amplification of lncRNA-*FAL1* was found to be associated with clinical outcomes in patients with ovarian cancer 5. Therefore, linking cancer-associated CNVs to lncRNAs will provide independent support for functional implications and lead to a greater understanding of cancer pathogenesis.

In its wild-type (WT) state, p53 is an important tumor suppressor and p53 pathway is activated in the presence of cellular stress, such as DNA damage and oncogenic signaling, and in turn coordinates the transcriptional response of hundreds of genes^6^. As a haplo-insufficient gene, a relatively small decrease of p53 level or activity can largely impact tumorigenesis 7. P53 activation can initiate multiple pathways that lead to a temporary pause at a cell-cycle checkpoint to allow for DNA repair, permanent growth arrest (senescence), or cell death (apoptosis) 8. Recently, Several molecules have been implicated in regulating p53 protein synthesis including translation initiation factors 9, RNA-binding proteins (RBPs) 10 and MDM2^11^. LncRNAs have been implicated in post-translational regulation of p53. For example, p53-induced lncRNA DINO can bind to p53 protein and promote its stabilization, regulating cell cycle arrest and apoptosis in response to DNA damage 12. While lncRNAs are known to be involved in p53 pathways, the role of lncRNAs in regulating the p53 protein remains mostly unknown.

In this study, we identify and characterize a novel long intergenic non-coding RNA PiHL (RP11-382A18.2). *PiHL*'s copy number amplification is significantly concurred with p53 protein downregulation without influencing its mRNA level. PiHL is upregulated in CRC and is associated with poor prognosis of CRC patients. Functional study reveals PiHL's role in maintaining CRC cell proliferation and inhibiting 5-FU induced apoptosis* in vitro* and *in vivo* in p53 wild type cancer cells. Mechanistically, PiHL acts to promote p53 ubiquitination by sequestering RPL11 from MDM2, through enhancing GRWD1 and RPL11 complex formation. Moreover, we show that PiHL is a transcriptional target of p53. Thus, our study has identified a novel lncRNA, PiHL, with a clinical, biological and mechanistic impact on human CRC.

## Methods

### Data collection

Gene expression, GISTIC (Genomic Identification of Significant Targets in Cancer) copy number alteration, RPPA (Reverse Phase Protein Arrays), and whole-exome mutation data were downloaded from TCGA Pan-Cancer Project. 23,117 genes, including 1,025 long non-coding intergenic RNAs and 18,706 protein coding genes, were annotated in 589 TCGA colorectal patient samples by GENCODE (v22, GRCh38).

### Data analysis

We used logarithmic mRNA expression data for further analysis. Spearman correlation analysis was used to analyze the correlation between the CNV and TP53 mRNA expression or p53 protein levels of 169 TP53 wild-type samples. Copy number frequencies of gain (CNV >= 1) or loss (CNV <= -1) were also computed. Fold changes of the gene expression between 644 tumors and 51 normal samples were calculated and the heatmap showing gene expression comparison was depicted by the z-score transformed expression profiles. We set 2 and 10-12 for the filter of the fold change and correlation between gene expression and CNV, respectively. Integrative Genome Browser (IGV) was used to delineate the copy number alterations in different regions.

### Patients and Specimens

Eighty-three matched primary cancer tissues and their corresponding adjacent noncancerous tissues were collected from colorectal cancer patients at Shanghai Jiao-tong University School of Medicine affiliated Tongren Hospital. These cases were selected based on a clear pathological diagnosis, and none of the patients had received preoperative anticancer treatment. Upon resection, human surgical specimens were immediately frozen in liquid nitrogen then stored at -80 ºC freezer for further investigation. Informed consent was obtained from each patient, and this study was approved by the Ethics Committee of Shanghai Jiao-tong University. Tissue microarray chips containing 100 pairs of colon cancer tissue samples matched to their adjacent noncancerous tissue samples and the associated clinicopathological information were purchased from Shanghai OUTDO Biotech Co. (Shanghai, China).

### Cell culture

SW620, LoVo, HT-29, SW480, HCT116, RKO CRC cell lines and HEK-293T cells were obtained from the Cell Bank of Type Culture Collection (Chinese Academy of Sciences, Shanghai, China). The isogenic p53-WT and p53-null HCT116 and RKO cells were previously generated by Bert Vogelstein's lab, Johns Hopkins University. Cells were maintained at 37 °C in a humidified incubator containing 5% CO_2_ in Dulbecco's modified Eagle medium (DMEM) (Gibco, Grand Island, NY, USA) supplemented with 10% fetal bovine serum (Gibco). All the cell lines were used within 20 passages and thawed freshly every 2 months. These cell lines were Mycoplasma-free and the genetic identity of the cell lines was confirmed by short tandem repeat (STR) profiling (ATCC).

### Guide RNA design and cloning

Briefly, the single guide RNA (sgRNA) expression vector lenti sgRNA (MS2)_puro backbone (Addgene, #73795) was digested with BsmBI and was gel-purified. A pair of 20 nt oligos containing the appropriate overhang was then ligated into the vector by mixing 1 μL of the cut vector (normalized to 100-200 ng/mL), 0.5 μL of each primer at a 100 mM stock concentration, 2 μL of 10x T4 DNA ligase buffer, and 1 μL of T4 DNA ligase (NEB #M0202T) into a total ligation volume of 20 μL. Ligations were left at room temperature overnight, and 1 μL of the ligation product was subsequently transformed into 10 μL of Stable 3 component cells. Resulting colonies were verified by Sanger sequencing.

### dCas9 activator plasmid construction

HCT116 cells were transduced with lenti dCAS-VP65_Blast (Addgene, #61425) and lenti MS2-P65-HSF1_Hygro (Addgene, #61426) simultaneously and cultured with high concentrations of Blasticidin (1 mg/mL) (Invivogen) and Hygromycin B (8 mg/mL) (Invivogen) for 14 days. The sgRNA expressing lentiviruses were generated in HEK293T as previously described. Supernatant containing viruses was collected 24-72 h after transfection. 72 h after transfection, stable HCT116 cells were infected with viruses then cultured in Puromycin (GIBCO) at a concentration of 1 ng/ml for 7 days.

### 5' and 3' rapid amplification of cDNA ends analysis

5'- and 3'-rapid amplification of cDNA ends (RACE) analyses were performed with 1 µg of total RNA or polyA+ RNA using the SMARTer RACE 5'/3' Kit (Clonetech, Palo Alto, CA, USA) according to the manufacturer's instructions^13^. The gene-specific primers (GSP), nested gene-specific primers (NGSP) and internal primers used for nested PCR are presented in Table S1.

### PiHL RNA Copy Number Analysis

Full-length PiHL was *in vitro* transcribed using Ribonucleotide solution set (NEB, Ipswich, USA) and T7 RNA polymerase (Roche, Mannheim, Germany), then treated with RNase-free DNase I (Promega, Madison, WI, USA) to digest DNA template. 0.5 μg total RNA or full-length PiHL RNA were synthesized into cDNA. Serial ten-fold dilutions (10^2^ to 10^9^ molecules per μl) of cDNA from *in vitro*-transcribed PiHL were used as reference molecules for standard curve calculation. Quantitative RT-PCR was performed as mentioned in Supplementary Methods.

### Subcellular fractionation

Separation of nuclear and cytosolic fractions was performed using the PARIS Kit (Ambion) according to the manufacturer's instructions. Cytoplasmic and nuclear fractions were split for RNA and protein extraction. The sequences for the primers are listed in Table S1.

### Isolation of Nucleoli

Nucleoli isolation in HCT116 cells was performed as described 14 with modification. 10^7^ HCT116 cells were suspended in 200 mL lysis buffer (10 mM Tris pH 8.0, 140 mM NaCl, 1.5 mM MgCl_2_, 0.5% NP-40, 2 mM RNase inhibitor), incubated on ice for 10 min. One tenth of the lysate was added to 1 mL Trizol for total RNA extraction. The rest of the lysate was centrifuged at 1200 g for 3 min at 4 °C to pellet the nuclei. Add 1 mL Trizol to the supernatant for cytoplasmic fraction extraction. To fractionate nuclear fractions, nuclei pellet was resuspended with 200 mL 340 mM sucrose solution containing 5 mM MgCl_2_. One tenth of the lysate was added to 1 mL Trizol for nuclei RNA extraction. To prepare nucleoplasmic and nucleolar fractions, nuclei were broken by sonication until intact nuclei cannot be detected in suspension by microscope. 200 mL 880 mM sucrose solution containing 5 mM MgCl_2_ was gently added to the bottom of sonicated nuclei and then centrifuged 20 min at 2,000 g, 4 °C to pellet nucleoli, and the supernatant was the nucleoplasmic fraction. Fractionated RNAs from the same number of cells were used for cDNA synthesis and RT-PCR.

### *In situ* RNA hybridization (ISH) for PiHL

Tissue microarray (TMA) chips of 100 paired colon cancers and normal tissues were incubated with double-DIG-labeled custom LNA probe for PiHL (5DigN-TTGGACACTGCATCAATAGTT-3DigN, Exiqon, Denmark) and detected with polyclonal anti-DIG Fab fragments (Roche, USA) and alkaline phosphatase conjugated secondary antibody (Invitrogen) using NBT-BCIP as the substrate. TMA were then counterstained with nuclear fast red staining solution (Sigma Chemical Co, USA). High-resolution images were captured with an Aperio Scan Scope AT Turbo (Aperio, Vista, CA, USA) equipped with Aperio Image Scope software (Aperio). Assessment of the staining was based on the staining intensity and the percentage of positively stained cells using Image-Pro Plus 6.0 software (Media Cybernetics, Inc., Silver Spring, MD, USA). The median signal of PiHL positive staining was defined as cutoff value.

### Biotin RNA pull-down assay

RNA pulldown assays were performed as previously described^13^. Biotinylated full-length PiHL and PiHL truncation probes were synthesized by T7 RNA polymerase using the Biotin RNA Labeling Mix (Thermo), and then incubated with the cell lysates for 1 h. Proteins with biotinylated PiHL were pulled down with streptavidin magnetic beads (Thermo) after incubation for 1 h. The samples were separated using SDS-PAGE and the specific bands were identified using mass spectrometry.

### RNA Immunoprecipitation

RNA Immunoprecipitation (RIP) experiments were performed using the Magna RIP RNA-Binding Protein Immunoprecipitation Kit (Millipore, Billerica, MA, USA), according to the manufacturer's instructions. Total RNA (input control) and precipitation with the isotype control (IgG) for each antibody were assayed simultaneously. The anti-GRWD1 antibody used for RIP were purchased from CST (Abcam, Cambridge, MA) (Table S2). The co-precipitated RNAs were pulled down by the protein G beads and then detected by qRT-PCR.

### *In vivo* tumor growth assays

Six-week-old male NOD/SCID mice were randomized into two groups (10 mice in each) and subcutaneously inoculated with 2 x 10^6^ HCT116 cells that stably expressed scrambled shRNA or PiHL shRNA (Table S3) in the right or left flanks, respectively. Tumor volume was calculated with the formula: tumor volume (mm^3^) = (length x width^2^)/2. Mice were sacrificed and tumors were harvested and weighed.

For 5-FU treatment xenograft models, 4-6 weeks old BALB/c nu/nu male athymic nude mice were randomized into different groups and inoculated (via subcutaneous injection) with HCT116 p53^+/+^ and HCT116 p53^-/-^ cells with or without ectopic PiHL expression (5 x 10^6^ cells in 0.2 ml PBS) for xenograft tumors formation. Tumor size was measured every 3 days with microcalipers in blind manner. When tumor volumes reached 200 mm^3^, mice were treated with 5-FU (i.p., 30 mg/kg daily) or vehicle for 12 days (6 mice in each group). All the *in vivo* experiments were performed according to our institution's guidelines for the use of laboratory animals and approved by the Committee on the Ethics of Animal Experiments of Fudan University.

### Immunoprecipitation

293T cells were transiently transfected with His-Xpress-MDM2 (1.2 μg), FLAG-RPL11 (1.8 μg), HA-GRWD1 (1 μg) and pCDH-PiHL (1 μg) as indicated for 48 h and cell extracts were prepared with NETN buffer (20 mM Tris-HCl [pH 8.0], 100 mM NaCl, 1 mM EDTA, 0.5% NP-40 and multiple protease inhibitors). Aliquots of the extracts were then immunoprecipitated with anti-FLAG antibody and Pierce Protein A/G Agarose (Thermo Fisher) overnight. Beads were washed with NETN buffer. The protein complex was then analyzed by western blotting.

### *In vitro* binding assay

Purified FLAG-RPL11 was incubated with purified HA-GRWD1, full-length PiHL or antisense PiHL and then bound to ANTI-FLAG M2 agarose beads (Sigma-Aldrich). After washing, the bound proteins were eluted and analyzed by immunoblotting.

### *In vitro* ubiquitination assay

*In vitro* ubiquitination assay was performed using a Human MDM2/HDM2 Ubiquitin Ligase Kit (R&D system, Minneapolis, Minnesota, USA). Purified His6-p53, GST-MDM2, FLAG-RPL11, HA-GRWD1 and biotin-PiHL were added as indicated. The samples were incubated with ubiquitin reaction components (E1, E2 and ubiquitin) at 37°C for 30 min to generate ubiquitinated p53 and subjected to SDS-PAGE followed by immunoblotting.

### RNA sequencing

HCT116 cells were transfected with siRNA-PiHL or siRNA-NC for 48 h before RNA isolation. Total RNA was sequenced using the Illumina HiSeq 3000 at Guangzhou RiboBio Co., Ltd. Raw reads were first filtered to remove the adaptor and bases of low quality by FASTX-Toolkit (Version 0.0.13). Filtered reads were aligned to human transcriptome hg19 by TopHat2 15 with the end-to-end method allowing two mismatches. Uniquely localized reads were then used to calculate read numbers and RPKM (reads per kilobase and per million) values for each gene according to reads and genes' genomic location. After obtaining the expression level of all genes in all the samples, differentially expressed genes were analyzed by using edgeR^16^. The expression data were deposited in GEO with an accession number.

### Statistical analysis

All statistical analyses were performed using SPSS software (Abbott Laboratories, Chicago, IL, USA) and GraphPad_Prism_7.0 (Graphpad Software Inc.). Survival curves were calculated using Kaplan-Meier and log-rank tests. The effects of variables on survival were determined by univariate and multivariate Cox proportional hazards modeling. The Mann-Whitney U test was used to analyze the relationship between PiHL expression and clinicopathologic characteristics. The Student t test was used to detect significance of data from qRT-PCR experiments and colony formation assays. Multiway classification analysis of variance test was performed for results from CCK8 assays and tumor growth curve determinations. All statistical tests were two-sided and P values were considered statistically significant for P ≤ 0.05.

Other details and additional experimental procedures are provided in Supplementary Methods.

## Results

### Identification of PiHL as a p53 protein regulator in CRC

**Figure 1:**
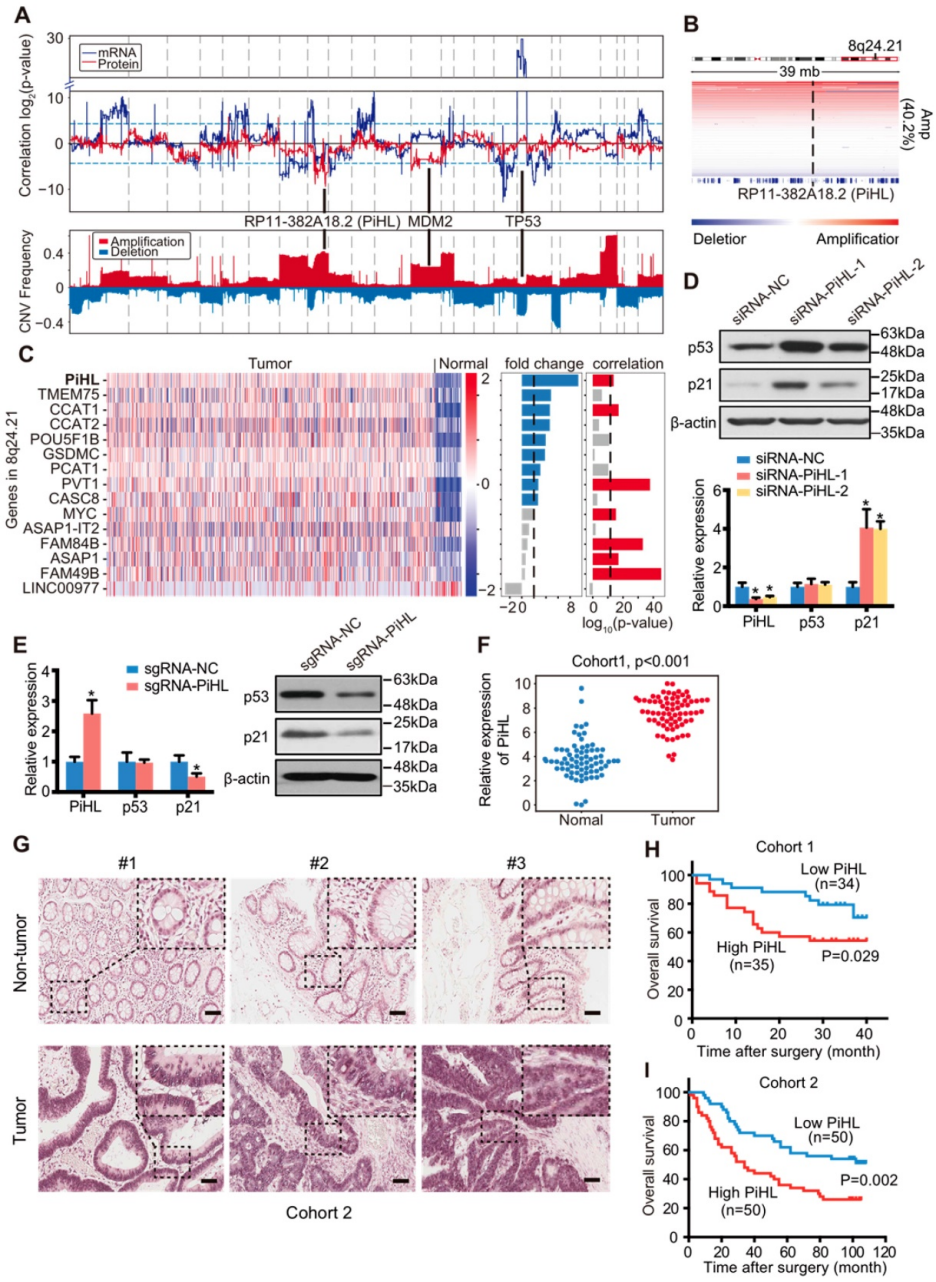
** Identification of p53 protein regulating lncRNAs in CRC.** (**A**) Upper panel: Correlation between genome-wide gene CNV and TP53 mRNA expression (blue line) or p53 protein (red line) level in 169 p53 wild-type samples. Lower panel: CNV frequency of copy number gain and loss in p53 wild-type samples. (**B**) IGV figures showing the copy number alterations of regions around PiHL in p53 wild-type samples. Amp: amplification. (**C**) Heatmap showing the gene expression in 466 tumors and 51 normal samples. Fold change of the gene expression of tumor versus normal, correlation of gene expression and its copy number are also plotted on the right of the heatmap. (**D**) Western blot and qRT-PCR analysis of p53, p21 and PiHL expression. HCT116 cells were transfected with siRNAs for PiHL or siRNA-NC. (**E**) Western blot and qRT-PCR analysis of p53, p21 and PiHL expression upon single guided RNA (sgRNA) transfection with the SAM system in HCT116 cells. (**F**) PiHL levels were quantified in 83 pairs of CRC tissues and adjacent normal tissues in cohort 1 using qRT-PCR. β-actin served as the control. Data are shown as mean ± s.e.m.; two-tailed Student's t-test. (**G**) Representative images of PiHL expression in CRC and adjacent tissues using ISH analysis in cohort 2; n = 100. Scale bar, 100 μm. (**H**) Kaplan-Meier analyses of the correlation between *PiHL* RNA levels and overall survival in cohort 1. (**I**) Kaplan-Meier analyses of the correlation between *PiHL* RNA levels and overall survival in cohort 2.

To identify lncRNAs that regulate p53 protein expression in CRC, we developed an intuitive strategy using whole-exome DNA sequencing (DNA-seq), genome-wide copy number alteration, RNA sequencing (RNA-seq) and RPPA data of 589 CRC tumors (Figure S1A). We reasoned that if the copy number alterations of some lncRNAs gene can influence p53 protein expression without changing TP53 mRNA levels, these lncRNAs may be involved in regulating p53 protein stability. Correlation analyses of the copy number altered lesions with p53 protein and mRNA expression in 169 p53-WT CRC tumors identified 24 regions correlated with p53 protein levels and 137 regions correlated with *TP53* transcription (Figure 1A and Table S4). The most prominent regions regulating *TP53* transcription is 17p13.1, where *TP53* gene is located (Figure 1A and Figure S1B). Consistent with previous report 17, *TP53* is deleted in 49 (29%) p53 WT CRC tumors and tumors with *TP53* deletion have significantly lower p53 mRNA expression (p=1.34×10^-7^, Figure 1A; Figure S1B and C). The coding gene of MDM2 protein, a well-established E3 ligase of p53 protein ubiquitination 18, is located in one of the top regions regulating p53 protein identified by our analysis (Figure 1A and Figure S1B). We observed that MDM2 was amplified in 47 (27.8%) CRC tumors, and tumors with *MDM2* amplification have significantly higher MDM2 mRNA expression (p=5.05×10^-7^, Figure 1A; Figure S1B and C). These data confirmed the capability of our approach to identify genomic copy number gains or losses in CRC, and to discover potential p53 regulators as the basis for further analysis.

Intriguingly, one of the most prominent regions negatively correlated with p53 protein in p53-WT but not p53 mutant samples is chromosome 8q24.21 (Figure 1A and B; Figure S1D). Chromosome 8q24.21 was reported to be frequently amplified in CRC and includes many lncRNA genes such as PVT1 and CCAT1 19-22. However, none of these genes have been reported to regulate p53 protein stability in CRC. RNA-seq data was applied to analyze the fold changes of genes located in 8q24.21 in CRC samples compared to normal samples. Among upregulated lncRNAs, the expression of CCAT1, PVT1 and a novel lncRNA RP11-382A18.2 were found to be significantly correlated with their copy number alteration (Figure 1C). Knockdown of these three lncRNAs using siRNAs showed that only silencing of RP11-382A18.2 strongly upregulated p53 protein levels but not mRNA expression in p53-WT HCT116 cells (Figure 1D; Figure S2A and B), thus we named this lncRNA PiHL (P53 inHibiting LncRNA). Since APC, KRAS, and TP53 mutations are often co-exist in colorectal cancer, we also filtered out APC or KRAS mutated CRC samples in p53 wildtype samples to eliminate possible confounding factors when analyzing PiHL CNV and p53 correlation. We found that in these samples, there was also a negative correlation trend, between p53 protein level and PiHL CNV, but not between PiHL CNV and TP53 (Figure S2C). To further confirm PiHL's regulation on p53 protein, we activated the endogenous transcription of *PiHL* gene using the CRISPR synergistic activation mediator (SAM) system 23. Consistently, activation of PiHL downregulated p53 protein but not p53 mRNA levels in HCT116 cells (Figure 1E). By expanding our analysis on other cancer types from TCGA database, we found that although PiHL was also upregulated in cancer types other than CRC, like stomach adenocarcinoma (STAD), cholangiocarcinoma (CHOL), bladder carcinoma (BLCA) and liver hepatocellular carcinoma (LIHC), correlation between PiHL copy number and p53/TP53 was not observed in these cancer types (Figure S2D). In aggregate, genomic, transcriptomic and proteomics analysis with functional screening identified lncRNA PiHL as a potential p53 protein regulator upregulated in CRC.

### LncRNA candidate PiHL is clinically relevant in CRC

PiHL is a long intergenic non-coding RNA located on human chromosome 8 (Figure S3A). RACE assay revealed that PiHL had three exons with a full length of 599 nt (Figure S3B). The transcript's non-coding nature was suggested by Coding Potential Assessing Tool (CPAT, Figure S3C) and lack of consistent open reading frames (ORFs) (ORF Finder, https://www.ncbi.nlm.nih.gov/orffinder/; Figure S3D).

We profiled PiHL expression in a panel of CRC cell lines (Figure S4A and B), separated the nuclear and cytoplasmic fractions of LoVo, HT-29, HCT116 and RKO cells, and measured PiHL's subcellular localization by qRT-PCR. In these samples, a considerable enrichment of PiHL was found in the nucleus versus the cytosol, indicating that PiHL is mainly localized in the nucleus (Figure S4C and D).

We next validated the upregulation of PiHL in 83 CRC tissues and paired adjacent normal tissues by qRT-PCR (Cohort 1, Figure 1F). *In situ* hybridization analyses of 100 independent paraffin-embedded CRC specimens confirmed the overexpression of PiHL in CRC tissues (Cohort 2, Figure 1G). Next, we analyzed the association between PiHL and clinicopathologic status in CRC patients from Cohort 1. A significant correlation was found between high levels of PiHL and poor tumor differentiation (p = 0.034), and large tumor size (p=0.020) (Table S5 and 6). A similar correlation between high levels of PiHL and large tumor size was observed in Cohort 2 (p=0.024 and Table S7 and 8). Furthermore, Kaplan-Meier survival analyses of Cohort 1 and 2 revealed that higher PiHL expression were significantly associated with poorer overall survival (OS) (p=0.029, Figure 1H; p=0.002, Figure 1I). Multivariate Cox regression analysis suggested that PiHL expression was independently correlated with CRC OS (Table S9 and 10). Taken together, these data suggest that increased PiHL levels are correlated with a poor prognosis in CRC patients.

### Effects of PiHL on p53 signaling and function in CRC cells

**Figure 2:**
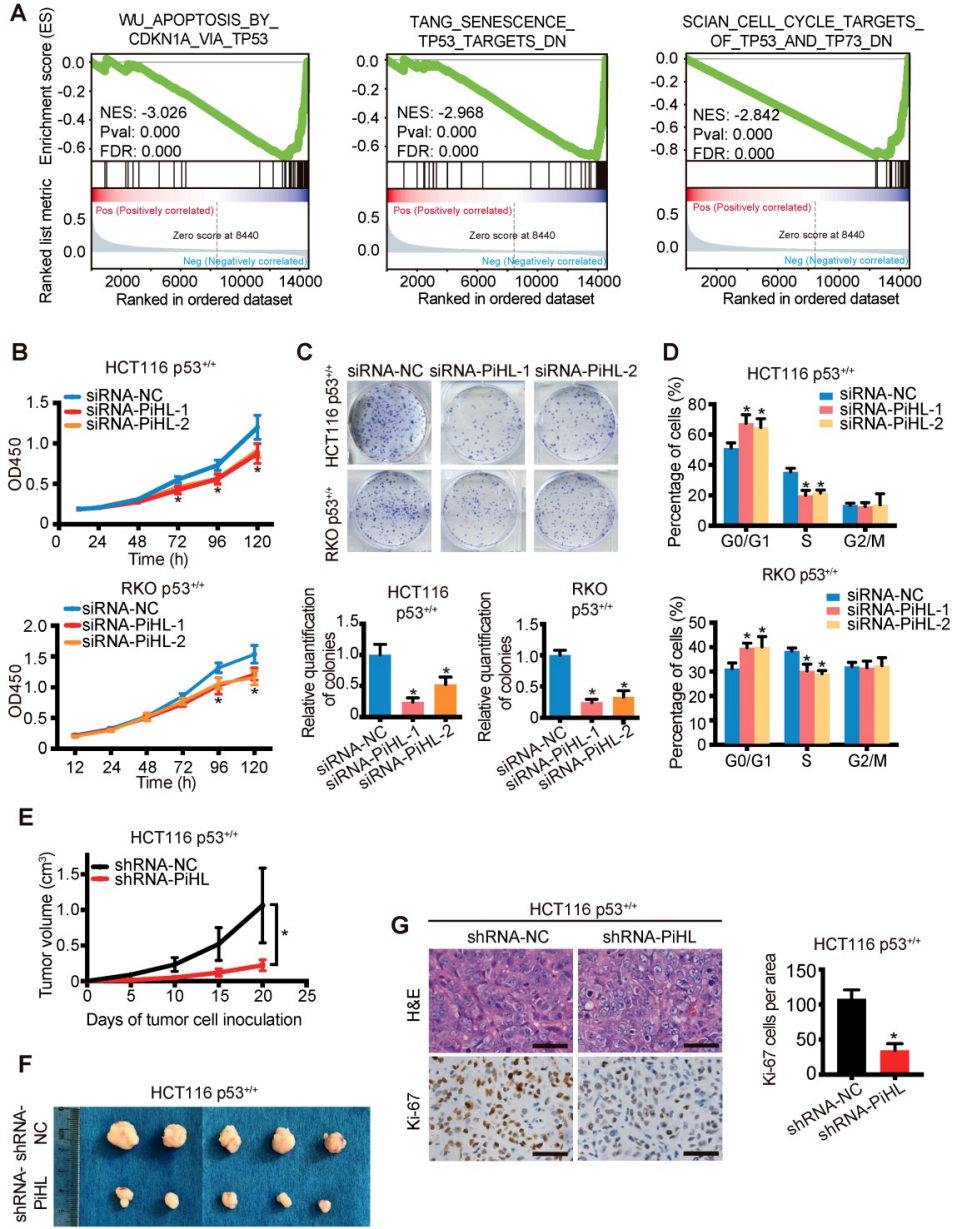
** PiHL promotes CRC tumorigenesis *in vitro* and* in vivo* in p53 WT cells.** (**A**) Gene set enrichment analysis (GSEA) results based on PiHL expression levels (siRNA-PiHL vs siRNA-NC, with three repeats) in HCT116 cells. The GSEA plots for the enrichment of p53 target genes involved in modulation of apoptosis and cell cycle are shown. (**B-D**) CCK-8 assays (**B**), colony formation assay (**C**), and cell-cycle analysis (**D**) in HCT116 p53^+/+^ and RKO p53^+/+^ cells upon the introduction of control siRNA or siRNAs (siRNA-PiHL-1 and siRNA-PiHL-2) against PiHL. (**E**, **F**) Quantification of tumor weight (**E**) and representative tumor size (**F**) from xenograft mouse models. (**G**) Left, Representative hematoxylin and eosin (H&E) and immunohistochemistry (IHC) staining of Ki-67 in tumors. Scale bars, 40 μm. Right, Ki-67 staining-positive cells were quantified as means ± s.e.m. for **B-E** and** G**, n = 3 for technical replicates. *p < 0.05.

Next, we sought to test the hypothesis that PiHL regulates the p53 pathways, as predicted by bioinformatics analysis. We manipulated PiHL's expression in HCT116 and RKO cells, respectively (Figure S5A and B). RNA-seq was performed to obtain the transcriptional profiles of HCT116 cells with PiHL knockdown (Table S11). GO and KEGG analysis revealed an enrichment of genes involved in multiple processes and important signaling pathways related to cancer (Figure S5C and D). Furthermore, gene set enrichment analysis (GSEA) of RNA-seq data indicated that p53 target genes involved in modulation of apoptosis and cell cycle were most prominently enriched in PiHL silencing cells (Figure 2A). Consistently, enforced PiHL expression led to a decrease of p53 protein and p53 target genes p21 and PUMA; whereas suppression of PiHL increased the accumulation of p53 protein, p21 and PUMA in p53 ^+/+^ HCT116 and RKO cells (Figure S6A-D) but not in p53 mutant HT-29 cells (Figure S6E and F), nor in p53 null HCT116 and RKO cells (Figure S6G-J), indicating that PiHL regulation of p53 pathway is dependent on WT p53. We noticed that another lncRNA, CASC8, is located at the same region but with opposite transcriptional direction with PiHL (Figure S3A). CASC8 expression remained unchanged after PiHL siRNA treatment (Figure S5A), suggesting that the phenotypic effects were not mediated by the siRNA off-target effect on CASC8.

We further determined whether PiHL plays an oncogenic role in a p53-dependent manner. Silencing of PiHL resulted in a strong anti-proliferative effect in p53^+/+^ CRC cells (Figure 2B and C). In contrast, the proliferative ability of CRC cells was increased by overexpression of PiHL (Figure S7A and B). Cell cycle analysis indicated that PiHL knockdown led to G1 arrest (Figure 2D); whereas the ratio of S phase cells was increased upon the transfection with PiHL (Figure S7C). Notably, in p53^-/-^ HCT116 and RKO cells, PiHL had less effect on regulating colon cancer cell viability (Figure S8A-D), and no effect on G1/S transition (Figure S8E and F).

Further, knockdown of PiHL by shRNA markedly delayed the growth of xenograft tumor derived from HCT116 p53^+/+^ cells than that from HCT116 p53^-/-^ cells (Figure 2E and F; Figure S9A). The stronger pro-proliferative effects of PiHL in p53^+/+^ cells than in p53^-/-^ cells were confirmed by Ki-67 expression (Figure 2G and Figure S9B). Collectively, these results demonstrate that PiHL plays a crucial role in cancer cell survival by predominantly suppressing the p53 pathway, though PiHL might also possess p53-independent functions in regulation of cell growth.

### PiHL physically interacts with GRWD1 and RPL11

**Figure 3:**
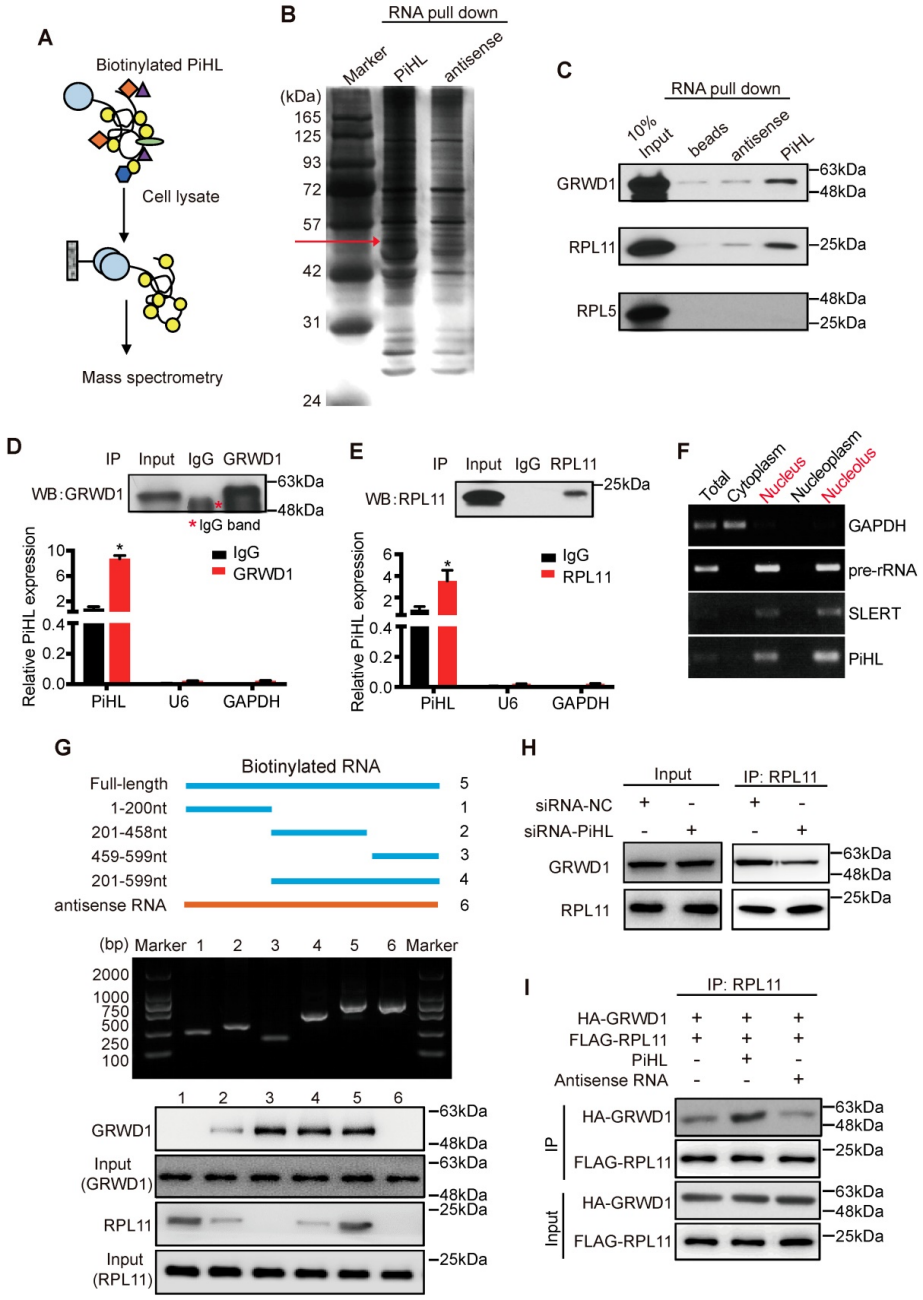
** PiHL interacts with GRWD1 and RPL11 complex.** (**A**) experimental design for pull-down assays and identification of PiHL-associated cellular proteins. PiHL and antisense-PiHL RNA were biotinylated by *in vitro* transcription, refolded, and incubated with HCT116 total cell lysates. (**B**) Silver staining of biotinylated PiHL-associated proteins. One PiHL-specific band (red arrow) was excised and analyzed by mass spectrometry. (**C**) Western blot of GRWD1, RPL11 and RPL5 proteins retrieved by* in-vitro*-transcribed biotinylated PiHL from PiHL cell nuclear extracts. Antisense PiHL and beads were used as negative controls. (**D, E**) HCT116 lysates were immunoprecipitated with anti-GRWD1 antibodies (**D**), anti-RPL11 antibody (**E**), or control IgG. Aliquots of cell lysates (20% of input) and IgG, GRWD1 or RPL11 immunoprecipitates were separated by SDS-PAGE, and the specific immunoprecipitation of GRWD1 and RPL11 was confirmed by Western blot (WB) (upper). The complexes were analyzed for the presence of PiHL, U6 or GAPDH by qRT-PCR (lower). Signals were normalized to actin mRNA. Results are mean ± s.e.m of three independent experiments. (**F**) PiHL accumulates to the nucleolus. Total RNA from HCT116 cells was separated into cytoplasmic, nuclear, nucleoplasmic, and nucleolar fractions and analyzed by RT-PCR. GAPDH RNA serves as a positive control for cytoplasmic gene expression, GAPDH and SLERT as positive controls for nucleolus separation. (**G**) Western blot of GRWD1 and RPL11 in samples pulled down by truncated (∆1: 1-200, ∆2: 201-458, ∆3: 459-599, ∆4: 201-599), full-length (5) or antisense (6) PiHL. (**H**) immunoprecipitation assay was performed to detect the interaction between GRWD1 and RPL11 after transfection of PiHL siRNA. The 20% of input (cell lysate) and RPL11 immunoprecipitates were confirmed by Western blot. (**I**) PiHL promoted the binding between recombinant GRWD1 and RPL11 *in vitro*. Data shown represent three independent experiments.

PiHL primarily localizes to the nucleus of CRC cells (Figure S4C and D), which suggests that PiHL may function by physically interacting with transcriptional factors, histone regulators, and other cellular factors. A biotin-labeled RNA PiHL pull-down assay followed by mass spectrometric analysis was performed to identify the proteins that might interact with PiHL (Figure 3A and B; Table S12). Among the potential proteins interacting with PiHL, GRWD1 has caught our attention. GRWD1, a WD40 protein that is highly conserved among eukaryotes, has been functionally implicated in ribosome biogenesis and tumorigenesis 24, 25. It has been reported to be overexpressed in cancer cells and its overexpression could down-regulate p53 levels through competitively binding with ribosomal proteins L11 (RPL11) 24, 26. Under ribosome biogenesis stress, several well-studied ribosomal proteins (RPs), including RPL5 and RPL11, have been shown to be released from the nucleolus, bind with MDM2 and inhibit its ubiquitin ligase activity toward p53, resulting in p53 accumulation^27^. We therefore explored whether lncRNA PiHL could also bind with RPL5 or RPL11, to regulate p53 levels. Further *in vitro* synthesized *PiHL* RNA pull-down assay revealed that *PiHL* RNA can interact with GRWD1 and RPL11, but not with RPL5 (Figure 3C). RNA immunoprecipitate (RIP) assay was performed to confirm that GRWD1 and RPL11 can precipitate endogenous *PiHL* RNA, respectively (Figure 3D and E). We detected approximately 9-fold and 3.5-fold enrichments of PiHL in the anti-GRWD1 and anti-RPL11 immunoprecipitates, respectively, compared with the IgG control (Figure 3D and E). GRWD1 and RPL11 are localized to nucleolus and are released into nucleoplasm upon nucleolar stress. Subcellular fractionation followed by RT-PCR in HCT116 cells revealed that lncRNA PiHL also mainly accumulated in the nucleolus (Figure 3F). Thus, PiHL may specifically bind with GRWD1 and RPL11 in CRC cells.

To further identify the GRWD1/RPL11-interacting region(s) in PiHL, we performed a deletion mapping pull-down assay based on PiHL's secondary structure (Figure S10A). The 3′ fragment and 5' fragment of PiHL were necessary for the interaction with GRWD1 and RPL11, respectively (Figure 3G). GRWD1 has two major functional domains: an acidic domain on the N-terminus and a WD40 domain on the C-terminus 24 (Figure S10B). Results from an RIP assay showed that GRWD1 fragments containing aa residues 76-150 (N3) could interact with PiHL (Figure S10B and C). Notably, PiHL depletion had no effect on the expression of GRWD1 or RPL11 (Figure S10D). However, PiHL knockdown diminished the relative amount of GRWD1 that associated with RPL11 in HCT116 cells (Figure 3H), indicating that PiHL promotes their interaction. Indeed, PiHL enhanced binding between purified GRWD1 and RPL11 in a cell-free system (Figure 3I). Thus, PiHL is important for GRWD1 and RPL11 complex interaction. Notably, silencing either GRWD1 or RPL11 could abrogate PiHL's regulation on p53, suggesting GRWD1 and RPL11 are required for PiHL's function via p53 pathway (Figure S10E).

### PiHL promotes p53 ubiquitination via GRWD1-RPL11 interaction

**Figure 4:**
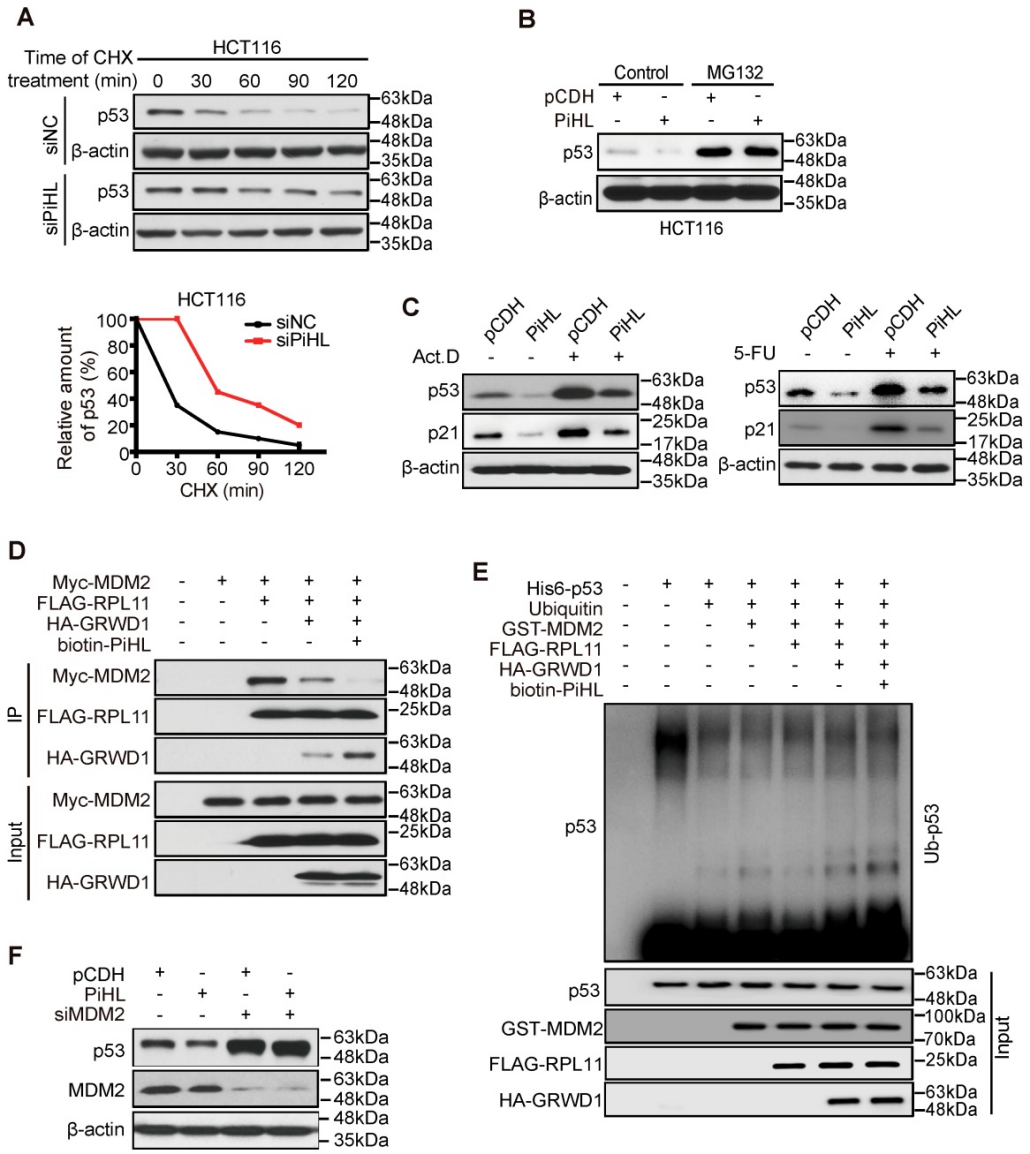
** PiHL inhibits p53 activity via MDM2-mediated ubiquitination.** (**A**) Half-life of p53 protein in PiHL-silencing (siPiHL) and control (siNC) HCT116 cells was shortened. Immunoblotting assays were used to detect p53 in HCT116 cells without or with the treatment of cycloheximide (CHX; 100 μg/mL). (**B**) MG132 (20 μM) abolishes the inhibitory effect of PiHL on p53 protein levels. (**C**) HCT116 cells with or without PiHL overexpression were treated with Act.D (5nM) or 5-FU (500 μM). The p53 and p21 proteins were examined by western blot assays. (**D**) Lysates were prepared from 293T cells co-transfected with Myc-MDM2 (1.2 μg), FLAG-RPL11 (1.8 μg), HA-GRWD1 (1 μg) and Biotin-PiHL (1 μg) as indicated for 48 h and then immunoprecipitated with anti-FLAG antibody. Immunoprecipitates (IPs) and inputs were immunoblotted with the indicated antibodies. (**E**) *In vitro* ubiquitination of p53 MDM2. Recombinant His_6_-p53 was incubated with E1, E2 (Ube2d3), ubiquitin, Mg^+^-ATP, FLAG-RPL11, HA-GRWD1, GST-MDM2 and biotinylated PiHL, or control immunoprecipitates were incubated at 37°C for 30 min as indicated. The samples were resolved by SDS-PAGE followed by immunoblotting with the indicated antibodies. (**F**) Knockdown of MDM2 attenuates the p53 degradation by PiHL overexpression.

Although p53 mRNA levels remained constant, its protein levels were drastically regulated when PiHL was silenced or overexpressed (Figure 1D, E and Figure S6A, B). This was attributable to proteasomal degradation as p53 levels were stabilized by proteasomal inhibitor MG132 (Figure 4A), and p53 stability increased by PiHL knockdown in cells treated with the protein synthesis inhibitor cycloheximide (CHX) (Figure 4B). These results indicate that the reduced p53 abundance by PiHL is not due to transcriptional effects.

p53 degradation is primarily regulated by protein ubiquitination mediated by E3 ligase MDM2^18^. Studies have reported that, under nucleolar stress, RPL11 is released from nucleolus and forms a complex with MDM2 to inhibit its E3 ligase function 28. We then treated CRC cells with Actinomycin D (Act.D) and 5-Fluorouracil (5-FU) to induce cell nucleolar stress. Knocking down PiHL expression in cells showed increased p53 protein levels and p53 target genes expression under nucleolar stress and under control condition (Figure 4C; Figure S11A and B). We thus hypothesized that PiHL could interact with GRWD1 and RPL11 and enhanced GRWD1/RPL11's effect on p53 ubiquitination. As shown in Figure 4D, we recapitulated that GRWD1 can compete with MDM2 in binding RPL11. Overexpression of PiHL further increased GRWD1/RPL11 binding and prevented co-precipitation of MDM2 with RPL11 (Figure 4D). An *in vitro* ubiquitination assay with recombinant proteins showed that GRWD1 prevented the RPL11-mediated inhibition of p53 ubiquitination levels (Figure 4E). As expected, the presence of PiHL further enhanced GRWD1's effect on promoting p53 ubiquitination (Figure 4E). Notably, when MDM2 was depleted, overexpression of PiHL did not affect the p53 protein levels (Figure 4F). These results suggested that PiHL promotes MDM2-mediated p53 ubiquitination and degradation through GRWD1-RPL11 interaction.

### PiHL promotes chemoresistance in CRC cells and colorectal xenograft tumors

**Figure 5:**
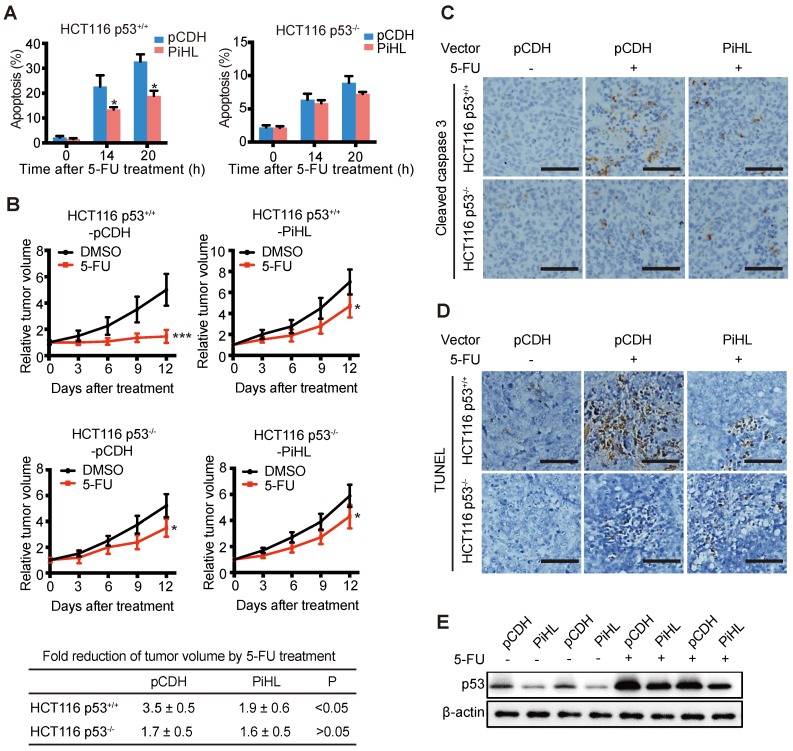
** PiHL promotes 5-FU resistance in colorectal HCT116 xenograft tumors.** (**A**) PiHL reduced p53-mediated apoptosis induced by 5-FU (500 μM) in p53^+/+^ and p53^-/-^ HCT116 cells. The percentage of apoptotic cells was determined by PI and Annexin V staining. (**B**) HCT116 p53^+/+^-PiHL, HCT116 p53^-/-^-PiHL and their control cells were employed for xenograft tumor formation in nude mice. When tumor volumes reached 200 mm^3^, mice were treated with 5-FU (30 mg/kg daily) or vehicle for 12 days. Relative tumor volumes are presented as mean±s.e.m, n=6. The fold reduction of tumor volumes by 5-FU for each group was calculated. *p < 0.05, ***p < 0.001 (**C, D**) 5-FU-induced apoptosis was determined by IHC staining of cleaved caspase 3 (**C**) and TUNEL assay (**D**) in xenograft tumors. Scale bar, 50 mm. (**E**) The levels of p53 protein were determined in HCT116 p53^+/+^ and HCT116 p53^-/-^ tumors treated with and without 5-FU.

Since we observed PiHL's regulation on p53 under 5-FU treatment, we sought to explore whether PiHL induce 5-FU resistance in CRC. Overexpression of PiHL displayed a drastic anti-apoptotic effect on p53^+/+^ CRC cells after 5-FU treatment (Figure 5A and Figure S12A), whereas PiHL had less effect on regulating 5-FU-induced apoptosis in p53 knockout HCT116 and RKO cells (Figure 5A and Figure S12A). Next, we further investigate whether downregulation of p53 by PiHL promotes chemoresistance *in vivo*. HCT116 p53^+/+^ and HCT116 p53^-/-^ cells with PiHL stable overexpression or controls were inoculated into BALB/c nude mice. When tumors reached 200 mm^3^, mice were then treated with 5-FU or vehicle for 12 days. As expected, 5-FU inhibited tumor growth in a largely p53-dependent way (Figure 5B). In response to 5-FU treatment, PiHL overexpression desensitized p53 wild type HCT116 tumor to 5-FU compared to control (Figure 5B). Average HCT116 p53^+/+^-pCDH tumor sizes reduced by 3.5-fold, whereas average HCT116 p53^+/+^-PiHL tumor sizes only reduced by 1.9-fold (Figure 5B). Notably, 5-FU therapeutic resistance promoted by PiHL was greatly reduced in HCT116 p53^-/-^ tumors (Figure 5B). Histological analyses of caspase 3 activation revealed that 5-FU treatment markedly increased cleaved caspase 3 levels in HCT116 p53^+/+^ tumors, but had a very limited effect in HCT116 p53^-/-^ tumors (Figure 5C). These results suggested that 5-FU induced p53-mediated apoptosis in HCT116 tumors. PiHL overexpression, in p53 wild type tumors, greatly inhibited apoptosis induced by 5-FU, but had limited effect in HCT116 p53^-/-^ tumors (Figure 5C). These results were further confirmed by TUNEL assay (Figure 5D). Consistent with the observation made in *in vitro* cultured cells, the levels of basal p53 protein and the accumulation of p53 protein in response to 5-FU treatment were much lower in HCT116 p53^+/+^-PiHL tumors than HCT116 p53^+/+^-pCDH tumors (Figure 5E). These results suggest that the downregulation of p53 protein levels and function is an important mechanism by which PiHL promotes chemoresistance in colorectal xenograft tumors.

### PiHL is a transcriptional target of p53

**Figure 6:**
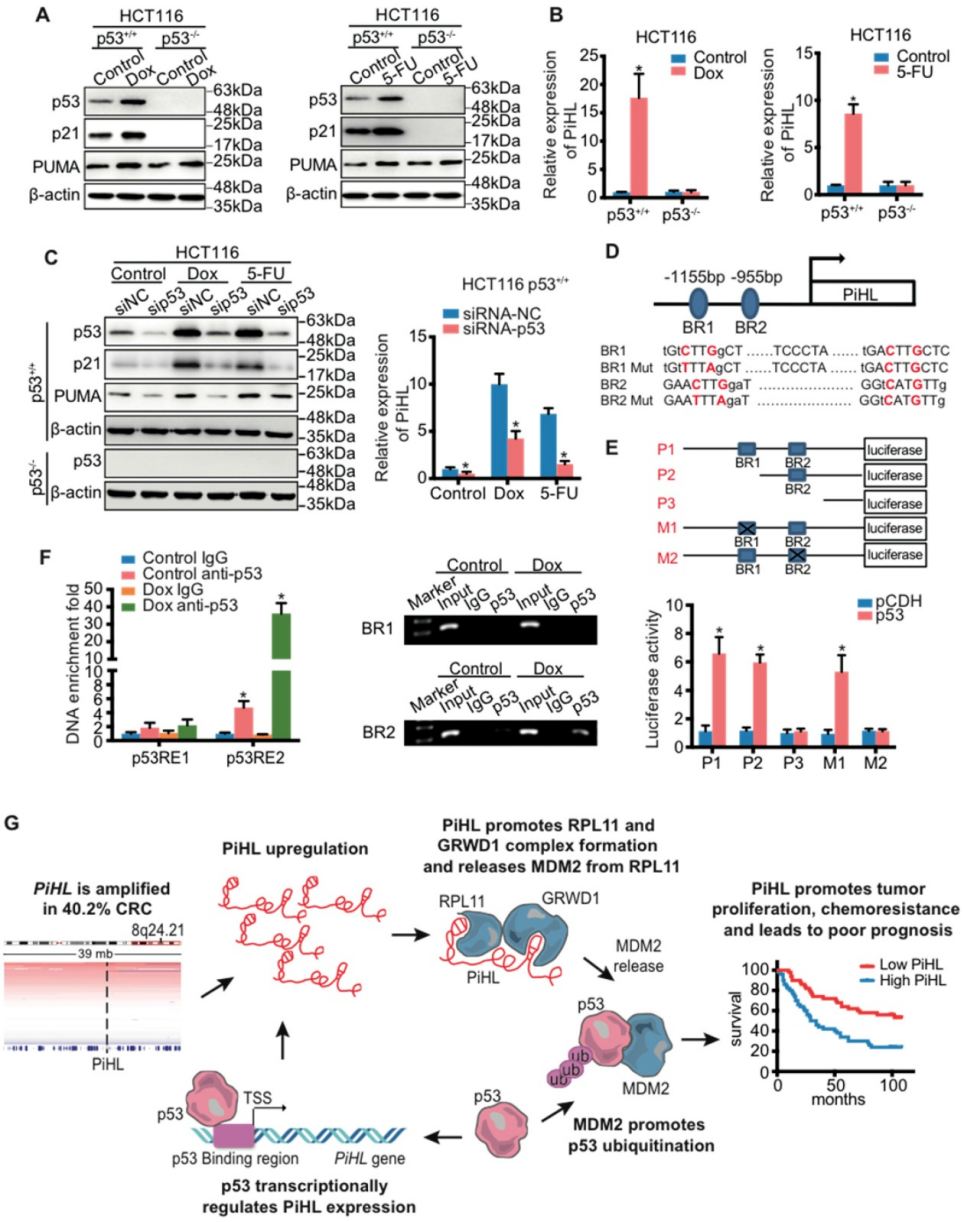
** PiHL is a direct transcriptional target gene of p53.** (**A, B**) HCT116 cells were treated with chemotherapy drugs Doxorubicin (Dox) (300 nM) or 5-Fluorouracil (5-Fu) (100 μM) for 20 h before analyses of RNA and protein levels. The protein levels of p53 and p53 targets were detected using immunoblotting analysis with indicated antibodies (**A**). PiHL levels were measured using RT-qPCR (**B**). (**C**) The effect of p53 knockdown on the protein levels and PiHL levels after treated cells with chemotherapy drugs. CRC cells were transfected with siRNA-NC or siRNA-p53 for 48 h, and treated with Dox or 5-Fu for 20 h before the cells were harvested for immunoblotting with indicated antibodies or RT-qPCR. β-actin served as the control. Data are shown as mean ± s.e.m.; two-tailed Student's t-test. (**D**) Two potential p53 binding regions (BR1 and BR2) were identified in the human PiHL promoter region using computer software (p53MH algorithm). The mutations of BR1 and BR2 were generated by site-directed mutagenesis. (**E**) Top: Truncation and mutation of PiHL promoter. P1 is a full-length promoter; P2 carries p53BR2; P3 carries neither p53BR1 nor p53BR2; M1 is a mutant at p53BR1 and M2 is a mutant at p53BR2. Bottom: Relative luciferase activity for corresponding constructs in HCT116 cells with p53 overexpression or controls. Error bars represent mean ± s.e.m, n = 3. *P < 0.05 by two-tailed t-test. (**F**) Confirmation of p53 binding to p53BR2 in PiHL promoter as detected by ChIP assay. HCT116 cells were treated with 1 mM Dox for 20 h, and ChIP assays were performed with the p53 antibody or control IgG. The promoter regions of indicated genes were analyzed by RT-qPCR. Error bars represent ±s.e.m, n = 3. *P < 0.05 by two-tailed t-test. (**G**) A proposed model of the functional consequence of PiHL overexpression in CRC tumorigenesis.

A previous study reported transcripts regulated upon Dox treatment in a p53-dependent manner using RNA-sequencing data 29. Their analysis revealed that PiHL could be highly induced along with well-established p53-regulated genes, including p21 and PUMA in p53 wild type HCT116 cells after DNA damage 29. To confirm this result, we exposed HCT116, RKO and HT-29 cells to Dox or 5-Fu and found that these chemotherapeutic drugs increased PiHL levels in WT p53-containing cells, but not in p53-null cells (Figure 6A, B and Figure S13A, B) or p53 mutant cells (Figure S13C and D). This result suggests that PiHL might be regulated by p53.

Indeed, knockdown of p53 in HCT116 p53^+/+^ and RKO p53^+/+^ cells resulted in a marked decrease of Dox- or 5-Fu- induced PiHL levels (Figure 6C and Figure S13E, F); whereas ectopic WT p53 induced PiHL expression (Figure S13G and H). We further cloned the two potential p53-binding regions (BR1 and BR2) at 1155 bp and 955 bp upstream, respectively, from the transcriptional initiation site of PiHL, and constructed corresponding luciferase reporters (Figure 6D and E). Ectopic p53 induced the activity of a luciferase reporter whose expression was driven by the PiHL promoter that contains BR2, but not BR1 or mutated BR2, suggesting that BR2 is likely the p53-binding DNA element in this promoter (Figure 6E). This result was further verified by ChIP assays (Figure 6F). Collectively, these data support the transactivation of PiHL by p53, acting through the identified p53-BR2.

## Discussion

In current study, we developed a strategy using TCGA data to identify copy number abnormalities that regulate p53 mRNA or protein expression. This strategy successfully recapitulated the well-established copy number regulation of p53 expression including *TP53* gene deletion and *MDM2* gene amplification. We also identified chromosome 8q24.21 as a region strongly correlated with p53 protein levels. Further computational and functional screening narrowed down the candidates to a novel lncRNA, PiHL, whose copy number amplification may mediate p53 protein stability. Using two independent cohorts, we have shown that PiHL expression is significantly associated with tumor size and CRC prognosis.

Given the importance of p53 in diverse cellular pathways, the cellular level of p53 protein is under tight regulation to maintain its proper activities and function in cells. P53 inactivation through mutation or deletion occurs in >50% of human cancers 30. Other mechanisms to deactivate p53 also exist. For example, the cell may express a number of p53 negative regulators, including MDM2, Pirh2 and LIF 31-33. Recently, lncRNAs have emerged as a group of regulators of p53 activity or expression. Amplification and/or overexpression of these p53 negative regulators have been frequently observed in tumors, which lead to the attenuation of p53 function and promotes tumorigenesis 34. Our study demonstrates that PiHL is a unique member of p53 negative regulators because it is capable of suppressing the cellular p53 level under normal conditions or under nucleolar stress. PiHL knockdown can elevate basal p53 to impair CRC cell proliferation and inhibit tumorigenicity *in vivo*. Overexpression of PiHL promotes 5-FU chemoresistance in cultured CRC cells and colorectal xenograft tumors in a largely p53-dependent manner. Thus, these data consistently point to the notion that high PiHL expression is a decisive factor underlying human CRC aggressiveness, and PiHL could be an important factor contributing to the chemoresistance in CRCs.

MDM2 is a major E3 ubiquitin ligase controlling p53 stability through the ubiquitin-proteasome pathway 35, 36. Recently, many studies have provided another insight into the regulatory network of MDM2-p53 via ribosomal proteins. Ribosomal proteins 11 (RPL11) has been reported to bind to MDM2 protein and increase the p53 protein stability by inhibiting the association between MDM2 and p53 27, 37, 38. Suppression of RPL11 by PICT1 causes p53 repression and promotes cell growth 28. GRWD1, a ribosomal/nucleolar protein involved in cellular regulatory pathways, is particularly associated with cell growth control 25, 39, 40. Recently, Kayama et al. demonstrated that GRWD1 is a novel oncogene 24. They identified GRWD1 as a RPL11 interactor and further found that GRWD1 interferes with the p53 activation pathway via the RPL11-MDM2 axis by interacting with RPL11 and sequestering it from MDM2 24.

Our study has contributed to the emerging concept that lncRNAs act as guides, decoys, or scaffolds to control cellular processes 41-44. We gained mechanistic clues into how PiHL functions to repress a subset of the p53 transcriptional response, by biochemical experiments that identified a specific interaction among PiHL, GRWD1 and RPL11. PiHL can enhance interaction between GRWD1 and RPL11 by acting as a potential modular scaffold in CRC cells. This notion is supported by five lines of evidence: (i) a previous report has classified GRWD1 and RPL11 as potential RNA binding proteins^45^, supporting the possibility that GRWD1 and RPL11 may interact with lncRNAs; (ii) PiHL directly binds with GRWD1 and RPL11 via its 3' and 5' domain, respectively; (iii) PiHL localized in the nucleolus of CRC cells; (iv) PiHL increases the binding of the GRWD1/RPL11 complex and subsequent promote p53 ubiquitination; (v) Overexpression of PiHL does not affect p53 protein levels when MDM2 was depleted. By serving as RNA scaffold, PiHL need both GRWD1 and RPL11 to regulate p53 pathway and CRC progression. It is also possible that other mechanism among PiHL/GRWD1/RPL11 interaction exists. For example, PiHL may bind with one of the proteins to induce a conformational change in binding domain that make it more accessible to another protein. Future structural characterization of the PiHL-GRWD1-RPL11 complex would help to find out whether PiHL serves not only a scaffold but also functions via other mechanism. It is generally considered that MDM2 ubiquitin ligase functions in nucleoplasm while RPL11 mainly exists in nucleoli. Therefore, even if PiHL promotes GRWD1 and RPL11 binding in nucleoli, it may only play limited role on influencing MDM2-p53 pathway. It is possible that under normal condition, PiHL regulates p53 protein, in part, in RPL11 and GRWD1-independent manner.

In current study, we observed that PiHL had some p53-independent oncogenic functions. Mutations of the p53 tumor suppressor are usually mutually exclusive with other cancer-promoting genetic hits leading to MDM2 deregulation. However, evidence in both human tumors and mouse models supports the notion that MDM2 oncogenic functions extend beyond p53 regulation 46. For example, some human tumors harbor both MDM2 overexpression and p53 mutations 47, and MDM2-overexpressing mice in a p53-null background exhibit an increased incidence of sarcomas relative to p53 knockout (KO) mice 48. This may help to explain why in p53^-/-^ cells, PiHL can also affect proliferation, although much less significant than in p53^+/+^ cells.

On top of demonstrating PiHL is a negative regulator of p53, we have further revealed the transcriptional regulation of PiHL by p53. Accumulating evidence suggests that, besides protein coding genes, many lncRNAs are also transcriptional targets of p53. For example, lincRNA-p21 could be activated in a p53-dependent manner in head and neck squamous cell carcinoma 49, 50. Hung et al. discovered that another p53 regulated lncRNA PANDA could interact with the transcription factor NF-YA to limit expression of pro-apoptotic genes in cancer cells 51. Among p53-regulated lncRNAs, many have been found to be p53 negative regulators, allowing them to form a negative feedback loop with p53 18, 29, 52. Like these regulators, PiHL is also under control of p53. This auto-regulatory feedback loop may signify a critical role of PiHL as an important p53 repressor. In physiological condition, an unwanted induction of p53 could be deleterious to the cell 53. Thus, the cell must exert precise control over the p53 level 54. One such control mechanism may involve PiHL-mediated suppression because the PiHL level is also increased when p53 is induced. The cancer cell may hijack this auto-regulatory feedback loop to the benefit of tumorigenesis. Indeed, we observed that PiHL is located at the most frequently amplified region (chr8q24.21) in CRC (Figure 6G).

Finally, our characterization of PiHL regulating p53 via a novel, MDM2-dependent mechanism may have clinical consequences, as PiHL overexpression lessens sensitivity to chemotherapy agents in CRC cells. Although p53 is inactivated in a majority of cancers, many tumors have intact p53 signaling, and therapeutic activation of p53 signaling through MDM2 inhibition is being investigated in clinical trials 18, 55. Since PiHL is amplified in cancer cells and can increase MDM2 activity, targeting PiHL as well as MDM2 to reactivate p53 is a potential therapeutic strategy to enhance chemosensitivity in CRC, especially in tumors with PiHL overexpression.

## Supplementary Material

Supplementary Figures and Tables S1-S3.

Supplementary Tables S4.

Supplementary Tables S5-10.

Supplementary Tables S11.

Supplementary Tables S12.

## Abbreviations

LncRNA: long noncoding RNA
CRC: colorectal cancer
CNVs: copy number variations
PiHL: P53 inHibiting LncRNA
TCGA: The Cancer Genome Atlas Program
PVT1: plasmacytoma variant translocation 1
CCAT1: colon cancer associated transcript 1
GRWD1: Glutamate Rich WD Repeat Containing 1
RPL11: ribosomal protein L11
MDM2: mouse double minute 2 homolog
5-FU: 5- Fluorouracil
Act.D: Dactinomycin
CHX: cycloheximide
qRT-PCR: quantitative reverse transcription polymerase chain reaction
RIP: RNA immunoprecipitation
ChIP: chromatin immunoprecipitation
RNA-ISH: RNA-In situ hybridization

## References

[1] Tang YC, Amon A (2013). Gene copy-number alterations: a cost-benefit analysis. Cell.

[2] Comprehensive molecular characterization of human colon and rectal cancer Nature. 2012; 487: 330-7.

[3] Weischenfeldt J, Dubash T, Drainas AP, Mardin BR, Chen Y, Stutz AM (2017). Pan-cancer analysis of somatic copy-number alterations implicates IRS4 and IGF2 in enhancer hijacking. Nat Genet.

[4] Uszczynska-Ratajczak B, Lagarde J, Frankish A, Guigo R, Johnson R (2018). Towards a complete map of the human long non-coding RNA transcriptome. Nat Rev Genet.

[5] Hu X, Feng Y, Zhang D, Zhao SD, Hu Z, Greshock J (2014). A functional genomic approach identifies FAL1 as an oncogenic long noncoding RNA that associates with BMI1 and represses p21 expression in cancer. Cancer cell.

[6] Vousden KH, Prives C (2009). Blinded by the Light: The Growing Complexity of p53. Cell.

[7] Bond GL, Hu W, Bond EE, Robins H, Lutzker SG, Arva NC (2004). A single nucleotide polymorphism in the MDM2 promoter attenuates the p53 tumor suppressor pathway and accelerates tumor formation in humans. Cell.

[8] Levine AJ, Oren M (2009). The first 30 years of p53: growing ever more complex. Nat Rev Cancer.

[9] Yang DQ, Halaby MJ, Zhang Y (2006). The identification of an internal ribosomal entry site in the 5'-untranslated region of p53 mRNA provides a novel mechanism for the regulation of its translation following DNA damage. Oncogene.

[10] Zhang J, Cho SJ, Shu L, Yan W, Guerrero T, Kent M (2011). Translational repression of p53 by RNPC1, a p53 target overexpressed in lymphomas. Genes Dev.

[11] Yin Y, Stephen CW, Luciani MG, Fahraeus R (2002). p53 Stability and activity is regulated by Mdm2-mediated induction of alternative p53 translation products. Nat Cell Biol.

[12] Schmitt AM, Garcia JT, Hung T, Flynn RA, Shen Y, Qu K (2016). An inducible long noncoding RNA amplifies DNA damage signaling. Nat Genet.

[13] Kong F, Deng X, Kong X, Du Y, Li L, Zhu H (2018). ZFPM2-AS1, a novel lncRNA, attenuates the p53 pathway and promotes gastric carcinogenesis by stabilizing MIF.

[14] Xing YH, Yao RW, Zhang Y, Guo CJ, Jiang S, Xu G (2017). SLERT Regulates DDX21 Rings Associated with Pol I Transcription. Cell.

[15] Kim D, Pertea G, Trapnell C, Pimentel H, Kelley R, Salzberg SL (2013). TopHat2: accurate alignment of transcriptomes in the presence of insertions, deletions and gene fusions. Genome Biol.

[16] Robinson MD, McCarthy DJ, Smyth GK (2010). edgeR: a Bioconductor package for differential expression analysis of digital gene expression data. Bioinformatics.

[17] Liu Y, Zhang X, Han C, Wan G, Huang X, Ivan C (2015). TP53 loss creates therapeutic vulnerability in colorectal cancer. Nature.

[18] Wade M, Li YC, Wahl GM (2013). MDM2, MDMX and p53 in oncogenesis and cancer therapy. Nat Rev Cancer.

[19] Tseng YY, Moriarity BS, Gong W, Akiyama R, Tiwari A, Kawakami H (2014). PVT1 dependence in cancer with MYC copy-number increase. Nature.

[20] Tomlinson I, Webb E, Carvajal-Carmona L, Broderick P, Kemp Z, Spain S (2007). A genome-wide association scan of tag SNPs identifies a susceptibility variant for colorectal cancer at 8q24.21. Nat Genet.

[21] Harismendy O, Frazer KA (2009). Elucidating the role of 8q24 in colorectal cancer. Nat Genet.

[22] Xiang JF, Yin QF, Chen T, Zhang Y, Zhang XO, Wu Z (2014). Human colorectal cancer-specific CCAT1-L lncRNA regulates long-range chromatin interactions at the MYC locus. Cell Res.

[23] Bester AC, Lee JD, Chavez A, Lee YR, Nachmani D, Vora S (2018). An Integrated Genome-wide CRISPRa Approach to Functionalize lncRNAs in Drug Resistance. Cell.

[24] Kayama K, Watanabe S, Takafuji T, Tsuji T, Hironaka K, Matsumoto M (2017). GRWD1 negatively regulates p53 via the RPL11-MDM2 pathway and promotes tumorigenesis. EMBO Rep.

[25] Gratenstein K, Heggestad AD, Fortun J, Notterpek L, Pestov DG, Fletcher BS (2005). The WD-repeat protein GRWD1: potential roles in myeloid differentiation and ribosome biogenesis. Genomics.

[26] Takafuji T, Kayama K, Sugimoto N, Fujita M (2017). GRWD1, a new player among oncogenesis-related ribosomal/nucleolar proteins. Cell Cycle.

[27] Zhang Y, Lu H (2009). Signaling to p53: ribosomal proteins find their way. Cancer cell.

[28] Sasaki M, Kawahara K, Nishio M, Mimori K, Kogo R, Hamada K (2011). Regulation of the MDM2-P53 pathway and tumor growth by PICT1 via nucleolar RPL11. Nat Med.

[29] Li XL, Subramanian M, Jones MF, Chaudhary R, Singh DK, Zong X (2017). Long Noncoding RNA PURPL Suppresses Basal p53 Levels and Promotes Tumorigenicity in Colorectal Cancer. Cell Rep.

[30] Vogelstein B, Lane D, Levine AJ (2000). Surfing the p53 network. Nature.

[31] Wade M, Wahl GM (2009). Targeting Mdm2 and Mdmx in cancer therapy: better living through medicinal chemistry?. Mol Cancer Res.

[32] Leng RP, Lin Y, Ma W, Wu H, Lemmers B, Chung S (2003). Pirh2, a p53-induced ubiquitin-protein ligase, promotes p53 degradation. Cell.

[33] Yu H, Yue X, Zhao Y, Li X, Wu L, Zhang C (2014). LIF negatively regulates tumour-suppressor p53 through Stat3/ID1/MDM2 in colorectal cancers. Nat Commun.

[34] Adriaens C, Standaert L, Barra J, Latil M, Verfaillie A, Kalev P (2016). p53 induces formation of NEAT1 lncRNA-containing paraspeckles that modulate replication stress response and chemosensitivity. Nat Med.

[35] Kruse JP, Gu W (2009). Modes of p53 regulation. Cell.

[36] Meek DW (2009). Tumour suppression by p53: a role for the DNA damage response?. Nat Rev Cancer.

[37] Lohrum MA, Ludwig RL, Kubbutat MH, Hanlon M, Vousden KH (2003). Regulation of HDM2 activity by the ribosomal protein L11. Cancer cell.

[38] Bhat KP, Itahana K, Jin A, Zhang Y (2004). Essential role of ribosomal protein L11 in mediating growth inhibition-induced p53 activation. EMBO J.

[39] Higa LA, Wu M, Ye T, Kobayashi R, Sun H, Zhang H (2006). CUL4-DDB1 ubiquitin ligase interacts with multiple WD40-repeat proteins and regulates histone methylation. Nat Cell Biol.

[40] Sugimoto N, Maehara K, Yoshida K, Yasukouchi S, Osano S, Watanabe S (2015). Cdt1-binding protein GRWD1 is a novel histone-binding protein that facilitates MCM loading through its influence on chromatin architecture. Nucleic Acids Res.

[41] Sun TT, He J, Liang Q, Ren LL, Yan TT, Yu TC (2016). LncRNA GClnc1 Promotes Gastric Carcinogenesis and May Act as a Modular Scaffold of WDR5 and KAT2A Complexes to Specify the Histone Modification Pattern. Cancer Discov.

[42] Hu WL, Jin L, Xu A, Wang YF, Thorne RF, Zhang XD (2018). GUARDIN is a p53-responsive long non-coding RNA that is essential for genomic stability. Nat Cell Biol.

[43] Jiang M, Zhang S, Yang Z, Lin H, Zhu J, Liu L (2018). Self-Recognition of an Inducible Host lncRNA by RIG-I Feedback Restricts Innate Immune Response. Cell.

[44] Zhang L, Yang Z, Trottier J, Barbier O, Wang L (2017). Long noncoding RNA MEG3 induces cholestatic liver injury by interaction with PTBP1 to facilitate shp mRNA decay. Hepatology.

[45] Baltz AG, Munschauer M, Schwanhausser B, Vasile A, Murakawa Y, Schueler M (2012). The mRNA-bound proteome and its global occupancy profile on protein-coding transcripts. Mol Cell.

[46] Yang JY, Zong CS, Xia W, Yamaguchi H, Ding Q, Xie X (2008). ERK promotes tumorigenesis by inhibiting FOXO3a via MDM2-mediated degradation. Nat Cell Biol.

[47] Cordon-Cardo C, Latres E, Drobnjak M, Oliva MR, Pollack D, Woodruff JM (1994). Molecular abnormalities of mdm2 and p53 genes in adult soft tissue sarcomas. Cancer Res.

[48] Jones SN, Hancock AR, Vogel H, Donehower LA, Bradley A (1998). Overexpression of Mdm2 in mice reveals a p53-independent role for Mdm2 in tumorigenesis. Proc Natl Acad Sci U S A.

[49] Jin S, Yang X, Li J, Yang W, Ma H, Zhang Z (2019). p53-targeted lincRNA-p21 acts as a tumor suppressor by inhibiting JAK2/STAT3 signaling pathways in head and neck squamous cell carcinoma. Mol Cancer.

[50] Huarte M, Guttman M, Feldser D, Garber M, Koziol MJ, Kenzelmann-Broz D (2010). A large intergenic noncoding RNA induced by p53 mediates global gene repression in the p53 response. Cell.

[51] Hung T, Wang Y, Lin MF, Koegel AK, Kotake Y, Grant GD (2011). Extensive and coordinated transcription of noncoding RNAs within cell-cycle promoters. Nat Genet.

[52] Chao T, Zhou X, Cao B, Liao P, Liu H, Chen Y (2016). Pleckstrin homology domain-containing protein PHLDB3 supports cancer growth via a negative feedback loop involving p53. Nat Commun.

[53] Polyak K, Xia Y, Zweier JL, Kinzler KW, Vogelstein B (1997). A model for p53-induced apoptosis. Nature.

[54] Purvis JE, Karhohs KW, Mock C, Batchelor E, Loewer A, Lahav G (2012). p53 dynamics control cell fate. Science.

[55] Khoo KH, Verma CS, Lane DP (2014). Drugging the p53 pathway: understanding the route to clinical efficacy. Nat Rev Drug Discov.

