# Supplementary material for: Long noncoding RNA PiHL regulates p53 protein stability through GRWD1/RPL11/MDM2 axis in colorectal cancer
Source: Theranostics. 2020 Jan 1;10(1):265–80. doi: 10.7150/thno.36045 (PMC6929633; doi:10.7150/thno.36045)
Supplement: Supplementary file 1 — Supplementary Figures and Tables S1-S3. [file thnov10p0265s1.pdf]

Supplementary Information for:

Long noncoding RNA PiHL regulates p53 protein stability through GRWD1/RPL11/MDM2 axis in colorectal cancer

Xuan Deng, Sihan Li, Fanyang Kong, Haoyu Ruan, Xiao Xu, Xinju Zhang, Zhiyuan Wu, Lin Zhang, Ying Xu, Hong Yuan, Haixia Peng, Da Yang, Ming Guan

Corresponding Authors:

Dr. Ming Guan, Email: guanming@shmu.edu.cn; Dr. Da Yang, Email: dyang@pitt.edu; Dr. Haixia Peng, Email: phx1101@shtrhospital.com.

This PDF includes:

Supplementary Methods

References

Supplementary Figure Legends

Supplementary Figure S1-12.

Supplementary Methods:

Antibodies and reagents

Information on antibodies used in this study is provided in Supplementary Table 2. Doxorubicin (Dox), 5-fluorouracil (5-FU), MG132 and cycloheximide (CHX) were purchased from MCE (Monmouth Junction, New Jersey, USA).

RNA isolation and quantitative RT-PCR analyses

RNA isolation was performed as previously described(1). Briefly, total RNA from the CRC tissue specimens and cell lines in this study was extracted using TRIzol reagent (Invitrogen, Carlsbad, CA, USA) or the RNeasy Mini Kit (Qiagen, Hilden, Germany), and reverse transcribed using PrimeScript™ RT reagent Kit with gDNA Eraser (Takara, Beijing, China). SYBR® Premix Ex Taq™ GC (Takara) was used for qPCR with primers listed in Supplementary Table 1. Expression levels were calculated relative to β-actin and normalized to control samples.

Plasmid construction and cell transfection

Full-length PiHL cDNA was amplified via PCR using the SeqAmp DNA Polymerase (Takara) and cloned into pCDH-CMV-puro (short for pCDH) vector (System Biosciences, Palo Alto, CA, USA). PiHL promoter was amplified from 2000 bp to 1 bp upstream of PiHL's transcription start site (TSS); the promoter truncations were amplified from 2000 bp, 1055 bp, or 615 bp to 1 bp upstream of PiHL's TSS. The promoter mutants were generated using the Q5® site-directed mutagenesis kit (NEB, Singapore) according to the manufacturer's instructions. These constructs of truncated or mutated PiHL promoter were subsequently cloned into the pGL3 vector at the *NheI* and *XhoI* sites.

Cells seeded on the plate overnight were transfected with plasmids as indicated in Figure legends using Lipofectamine® 3000 (Thermo Fisher Scientific, Waltham, MA, USA) transfection reagent following the manufacturer's protocol. Cells were harvested at 48-72 h post-transfection for future experiments.

RNA interference

SiRNA oligonucleotides targeting PiHL, PVT1, CCAT1, p53 and MDM2 are listed in Supplementary Table 3 (Biotend, Shanghai, China). Cells were transfected with the indicated siRNAs using Lipofectamine 3000 Reagent (Invitrogen) or Lipofectamine RNAiMAX Reagent (Invitrogen), according to the manufacturer's protocol. After transfection for 48 h, the cells were used for RNA extraction, CCK8, flow cytometry, apoptosis and immunoblotting assays. ShRNAs for PiHL were purchased from Biotend.

Western Blot

Western blot analysis was performed as previously described(2). Cells were suspended in RIPA lysis buffer (Beyotime, Shanghai, China) containing protease inhibitor cocktail (Sigma-Aldrich). Cell lysates or retrieved proteins were analyzed by immunoblot with primary antibodies and HRP-conjugated secondary antibodies.

Cell proliferation assays

The viability of CRC cells was determined by Cell Counting Kit 8 (CCK8; Dojindo Corporation, Kumamoto, Japan) as previously described(1). Approximately 1×10^3 transfected cells in 100 μl were incubated in triplicate in 96-well plates. At 0, 12, 24, 48, 72 and 96 h, the CCK-8 reagent (10 μl) was added to each well and incubated at 37 °C for 1 h. The optical density at 450 nm was measured using an automatic microplate reader (BioTek, Winooski, VT, USA).

Colony formation assay

HCT116 and RKO cells were seeded into 6-well plates at a density of 1×10^3 and 500 cells per well, respectively. After two weeks, cells were fixed by 100% methanol and stained with 0.1% crystal violet. Colonies were counted using Image J software (NIH, Bethesda, MD, USA).

Flow cytometry analysis

For the cell cycle analysis, 48 h after transfection, 1×10^6 cells in the log phase of growth were harvested by trypsinization, washed twice with cold PBS, fixed in ice-cold 70% ethanol, and incubated overnight at −20°C. Propidium iodide (PI, 50 ug/ml, Sigma) and RNaseA (0.1 mg/ml, Sigma) were added to the cells and stained for 15 min. Cell cycle profiles were captured using a FACS Calibur flow cytometer (BD, Biosciences, CA, USA), and the data were analyzed using ModFit LT software (Verity Software House, Inc., Topsham, ME, USA).

For the cell apoptosis assay, an Annexin V-FITC Apoptosis Detection Kit II (BD, Biosciences, CA, USA) was used by measuring the membrane redistribution of phosphatidylserine. Cells were treated according to the manufacturer's instructions. The pre-labeled cells were detected and apoptosis was quantified using a FACS Calibur flow cytometer with Cell-Quest software (BD Biosciences). Annexin V-FITC and PI double stain was used to evaluate the percentages of apoptosis. Annexin V− and PI− cells were used as controls. Annexin V+ and PI− cells were designated as apoptotic and Annexin V+ and PI+ cells were designated as necrotic. Each test was repeated in triplicate.

Terminal deoxynucleotidyl transferase-mediated nick-end labelling (TUNEL) assay

Apoptotic cells were detected by terminal deoxynucleotidyl transferase-mediated deoxyuridine triphosphate nick-end labeling (TUNEL) staining using an In Situ Apoptosis Detection Kit (Takara Bio Inc., Shiga, Japan) according to the manufacturer's instructions. The sections were counterstained with Mayer's hematoxylin and TUNEL-positive cells were quantified in 3 randomly selected fields of each section at ×400 magnification.

Luciferase assays

HEK-293T cells were seeded in 96-well plates at a density of 5,000 cells per well 24 h before transfection. The cells were co-transfected with a mixture of 45 ng PGL3-basic-PiHL promoter, 5 ng pRL-TK and 150 ng pCDH-p53 or control according to recommended instructions using Lipofectamine 3000 (Invitrogen). Twenty-four h after transfection, Firefly and Renilla luciferase activity was measured by the Dual-Luciferase Reporter Assay System (Promega, San Luis Obispo, CA, USA). Relative firefly luciferase activities were detected by a BD Monolight 3010 luminometer (BD Biosciences), and Renilla luciferase activities served as an internal control. The sequences for the primers are listed in Supplementary Table 1.

Chromatin immunoprecipitation (ChIP)

Cells were exposed to Dox or DMSO for 24h. Subsequently, ChIP assays were performed on chromatin extracts from these cells according to the manufacturer's specifications (EZ-ChIP kit-Millipore, Boston, MA, USA) with the following antibodies: rabbit anti-p53 (Abcam, Cambridge, UK) and normal rabbit IgG (Millipore, Boston, MA, USA). The immunoprecipitated DNA was purified and bound regions were identified by PCR analysis with primers (Supplementary Table 1) specific for the protein binding regions (BRs) within the promoter of PiHL.

URLs

The coding potential of the PiHL transcript was analyzed using the Coding Potential Assessment Tool (CPAT, http://lilab.research.bcm.edu/cpat/), Open Reading Frame Finder (https://www.ncbi.nlm.nih.gov/orffinder/) and the coding potential calculator (CPC, http://cpc.cbi.pku.edu.cn/). The PiHL promoter was analyzed in the JASPAR database (http://jaspar. genereg.net/).

Reference

1. Hu F, Deng X, Yang X, Jin H, Gu D, Lv X*, et al.* Hypoxia upregulates Rab11-family interacting protein 4 through HIF-1alpha to promote the metastasis of hepatocellular carcinoma. Oncogene **2015**;34(49):6007-17 doi 10.1038/onc.2015.49.
2. Kong F, Kong X, Du Y, Chen Y, Deng X, Zhu J*, et al.* STK33 Promotes Growth and Progression of Pancreatic Cancer as a Critical Downstream Mediator of HIF1alpha. Cancer research **2017**;77(24):6851-62 doi 10.1158/0008-5472.Can-17-0067.
3. Xing YH, Yao RW, Zhang Y, Guo CJ, Jiang S, Xu G*, et al.* SLERT Regulates DDX21 Rings Associated with Pol I Transcription. Cell **2017**;169(4):664-78.e16 doi 10.1016/j.cell.2017.04.011.
4. Kim D, Pertea G, Trapnell C, Pimentel H, Kelley R, Salzberg SL. TopHat2: accurate alignment of transcriptomes in the presence of insertions, deletions and gene fusions. Genome biology **2013**;14(4):R36 doi 10.1186/gb-2013-14-4-r36.
5. Robinson MD, McCarthy DJ, Smyth GK. edgeR: a Bioconductor package for differential expression analysis of digital gene expression data. Bioinformatics (Oxford, England) **2010**;26(1):139-40 doi 10.1093/bioinformatics/btp616.

Supplementary Figure Legends

Supplementary Figure 1. Identification of p53 regulating lncRNAs in CRC. (**A**) Schematics of the analysis performed to evaluate the association between copy number variations and TP53 expression/p53 level and identify the p53 regulating genes. (**B**) IGV figures showing the copy number alterations of regions around TP53 and MDM2 in p53 wild-type samples. Del: Deletion; Amp: amplification. (**C**) Left: TP53 mRNA differential expression between TP53 copy number deletion and other samples. ($p < 0.001$, Wilcoxon rank-sum test), y-axis represents the log2 transformed expression level. Right: MDM2 mRNA differential expression between MDM2 copy number amplification and other samples. ($p < 0.001$, Wilcoxon rank-sum test), y-axis represents the log2 transformed expression level. (**D**) Correlation between genome-wide gene CNV and TP53 mRNA expression (blue line) /p53 protein (red line) level (upper panel) and CNV frequency of copy number gain and loss (lower part) in TP53 mutated samples.

Supplementary Figure 2. PiHL's regulation on p53 protein in CRC. (**A, B**) Western blot and qRT-PCR analysis of p53, p21, PVT1 and CCAT1 expression. β-actin served as the control. Data are shown as mean ± s.e.m.; two-tailed Student's t-test. (**C**) Correlation of PiHL CNV and its protein expression (left) or its mRNA expression (right) are shown in APC, KRAS and TP53 wildtype CRC samples. Rho: correlation coefficient. (**D**) Correlation between PiHL copy number variations and p53 protein/TP53 mRNA expression in both TP53 wild-type and mutated samples in each cancer type are shown in the left, the color indicates the Spearman's rank correlation coefficient (rho), only significant correlations are shown with color. Log2(fold change) of PiHL expression between tumor versus normal tissues in each cancer type is shown in the right panel. Student's t-test was done to compare the expression difference and color in each bar indicates the significance level (-log10 p-value) of the t-test. The cancer types with more than 5 tumor samples and 5 normal samples are included here and are ranked by the fold change of PiHL expression in tumor versus normal.

Supplementary Figure 3. Cloning the full-length human PiHL gene. (**A**) Schematic representation of the PiHL and CASC8 locus. (**B**) Representative image of PCR products from the 5'-RACE, 3'-RACE and internal PCR (left), and the full-length PCR of PiHL (right) are shown. A red arrow on the right panel marks the major PCR product. The PiHL sequence is shown at the bottom. (**C**) PiHL was calculated to be a lncRNA with a CPAT coding probability of 0.0168. (**D**) ORF Finder software predicted that PiHL is an lncRNA.

Supplementary Figure 4. Expression and localization of PiHL in CRC cells. (**A**) Expression levels

of PiHL in different CRC cell lines were tested by qRT-PCR. β-actin served as the control. Data are shown as mean ± s.e.m. (**B**) Quantification of *PiHL* RNA copy number/cell according to a standard curve of *in-vitro* transcribed PiHL. (**C**) Expression of PiHL in cytoplasmic and nuclear fractionations of CRC cells. U6 RNA serves as a positive control for nuclear gene expression and GAPDH as a positive control for cytoplasmic gene expression. Data are shown as mean ± s.e.m. (**D**) RT-PCR analysis of PiHL expression levels in different subcellular fractionation of HCT116 cells treated with PiHL siRNAs. Cell fractionation was further confirmed by western blot using cytoplasmic marker (GAPDH) and nuclear marker (SNRP70).

Supplementary Figure 5. PiHL regulates genes were investigated by RNA-seq. (**A**) qRT-PCR analysis of PiHL and CASC8 in HCT116 and RKO cells treated with control siRNA or siRNAs (siRNA1 and siRNA2) against PiHL. (**B**) qRT-PCR analysis of PiHL in HCT116 and RKO cells treated with pCDH-PiHL (PiHL) or empty vector (pCDH). (**C, D**) Functional annotation clustering of genes regulated by PIHL depletion in HCT116 cells. Enriched groups listed by their gene ontology (GO) term (**C**) and Kyoto Encyclopedia of Genes and Genomesare (KEGG) term (**D**) are ranked on the basis of the significant enrichment scores.

Supplementary Figure 6. PiHL negatively regulates wild type p53 protein and target genes. (**A-J**) Western blot and qRT-PCR analysis of p53, p21, PUMA and PiHL expression in HCT116 $p53^{+/+}$, RKO $p53^{+/+}$, HT-29 (with mutant p53), HCT116 $p53^{-/-}$ and RKO $p53^{-/-}$ cells. Cells were transfected with pCDH-PiHL (PiHL)/vector plasmid (pCDH) or siRNA-PiHL/siRNA-NC and harvested 48h post transfection for immunoblotting with indicated antibodies (**A, C, E, G** and **I**) or qRT-PCR (**B, D, F, H** and **J**). β-actin served as the control. Data are shown as mean ± s.e.m.; *P<0.05 by two-tailed Student's t-test.

Supplementary Figure 7. PiHL promotes CRC cells growth *in vitro*. (**A-C**) CCK-8 assays (**A**), colony formation assay (**B**) and cell-cycle analysis (**C**) in HCT116 $p53^{+/+}$ and RKO $p53^{+/+}$ cells transfected with pCDH-PiHL (PiHL) or empty vector (pCDH). Error bars represent ±s.e.m, n = 3. *P < 0.05 by two-tailed t-test.

Supplementary Figure 8. Proliferation, Cell cycle and apoptosis analysis of $p53^{-/-}$ CRC cells. (**A-F**) CCK-8 assays (**A, C**), colony formation assay (**B, D**), and cell-cycle analysis (**E, F**) in HCT116 $p53^{-/-}$ and RKO $p53^{-/-}$ cells. Cells were transfected with pCDH-PiHL (PiHL)/vector plasmid (pCDH) or siRNA-PiHL/siRNA-NC and harvested 48h before further experiments.

Supplementary Figure 9. PiHL promotes CRC cell growth *in vivo* in p53-dependent manner. (**A**) Quantification of tumor weight and representative tumor size from HCT116 $p53^{-/-}$ xenograft mouse models. (**B**) Left, Representative hematoxylin and eosin (H&E) and immunohistochemistry (IHC) staining of Ki-67 in tumors. Scale bars, 40 μm. Right, Ki-67 staining-positive cells were quantified as means ± s.e.m. n = 3 for technical replicates. *p < 0.05.

Supplementary Figure 10. PiHL promotes GRWD1 and RPL11 interaction. (**A**) Secondary structure of PiHL analyzed by LNCipedia (http://www.lncipedia.org). (**B**) Schematic diagram of the plasmids encoding Flag-tagged full-length or the fragments of GRWD1 (upper panel). Lower panel: plasmids encoding Flag-tagged full-length or fragments of GRWD1 were transfected in the HCT116 cells, and their expression levels were detected by western blot. (**C**) RIP of each fragment was performed using the anti-Flag antibody, and qRT-PCR was used to determine the enriched levels of PiHL in the immunoprecipitates. Normal HCT116 cells were used as control. (**D**) Expression levels of GRWD1 and RPL11 were determined by western blot in PiHL depletion HCT116 cells or control cells. β-actin served as the internal control. (**E**) Knockdown of GRWD1 attenuates the p53 degradation by PiHL overexpression (left), silencing RPL11 attenuates the p53 accumulation by PiHL knockdown (right).

Supplementary Figure 11. PiHL negatively regulates p53 signaling under nucleolar stress. (**A, B**) HCT116 cell with or without PiHL overexpression were treated with Act.D (5 nM) (**A**) or 5-FU (500 μM) (**B**), the mRNA levels of p21 and PUMA were determined by qRT-PCR and normalized with β-actin. Error bars represent ±s.e.m, n = 3. *P < 0.05 by two-tailed t-test.

Supplementary Figure 12. PiHL regulates 5-FU induced apoptosis of RKO cells. (A) PiHL reduced p53-mediated apoptosis induced by 5-FU (500 μM) in $p53^{+/+}$ and $p53^{-/-}$ RKO cells. The percentage of apoptotic cells was determined by PI and Annexin V staining.

Supplementary Figure 13. PiHL is regulated by p53. (**A-D**) RKO and HT-29 cells were treated with chemotherapy drugs Doxorubicin (Dox) (300 nM) or 5-Fluorouracil (5-Fu) (100 μM) for 20 h before analyses of RNA and protein levels. The protein levels of p53 and p53 targets were detected using immunoblotting analysis with indicated antibodies. The PiHL levels were measured using RT-qPCR. Data are presented as mean ± s.e.m. n = 3 for technical replicates. *p < 0.05. (**E, F**) The effect of p53 knockdown on the protein levels (**E**) and PIHL levels (**F**) after treated cells with chemotherapy drugs.

CRC cells were transfected with siRNA-NC or siRNA-p53 for 48 h, and treated with Dox or 5-Fu for 20 h before the cells were harvested for immunoblotting with indicated antibodies or RT-qPCR. (**G, H**) The effect of p53 overexpression on PiHL levels. CRC cells were transfected with p53 or empty vector and harvested 48 h post-transfection. The p53 and p53 targets levels were determined by immunoblotting with corresponding antibodies (**G**). The relative PiHL level was quantified by RT-qPCR (**H**). β-actin served as the control. Data are shown as mean ± s.e.m., n = 3. *P < 0.05 by two-tailed t-test.

Supplementary Figure 1

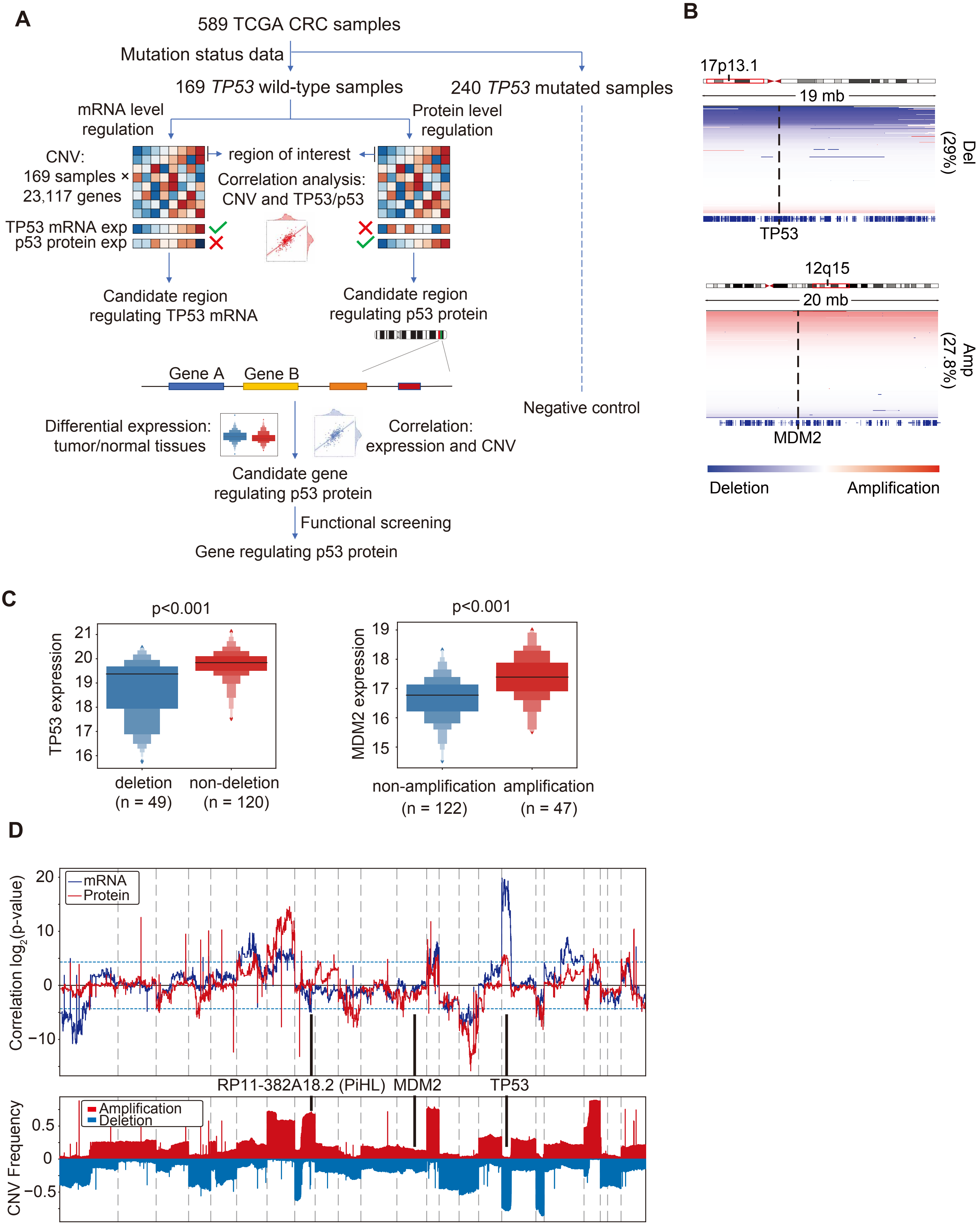

Supplementary Figure 2

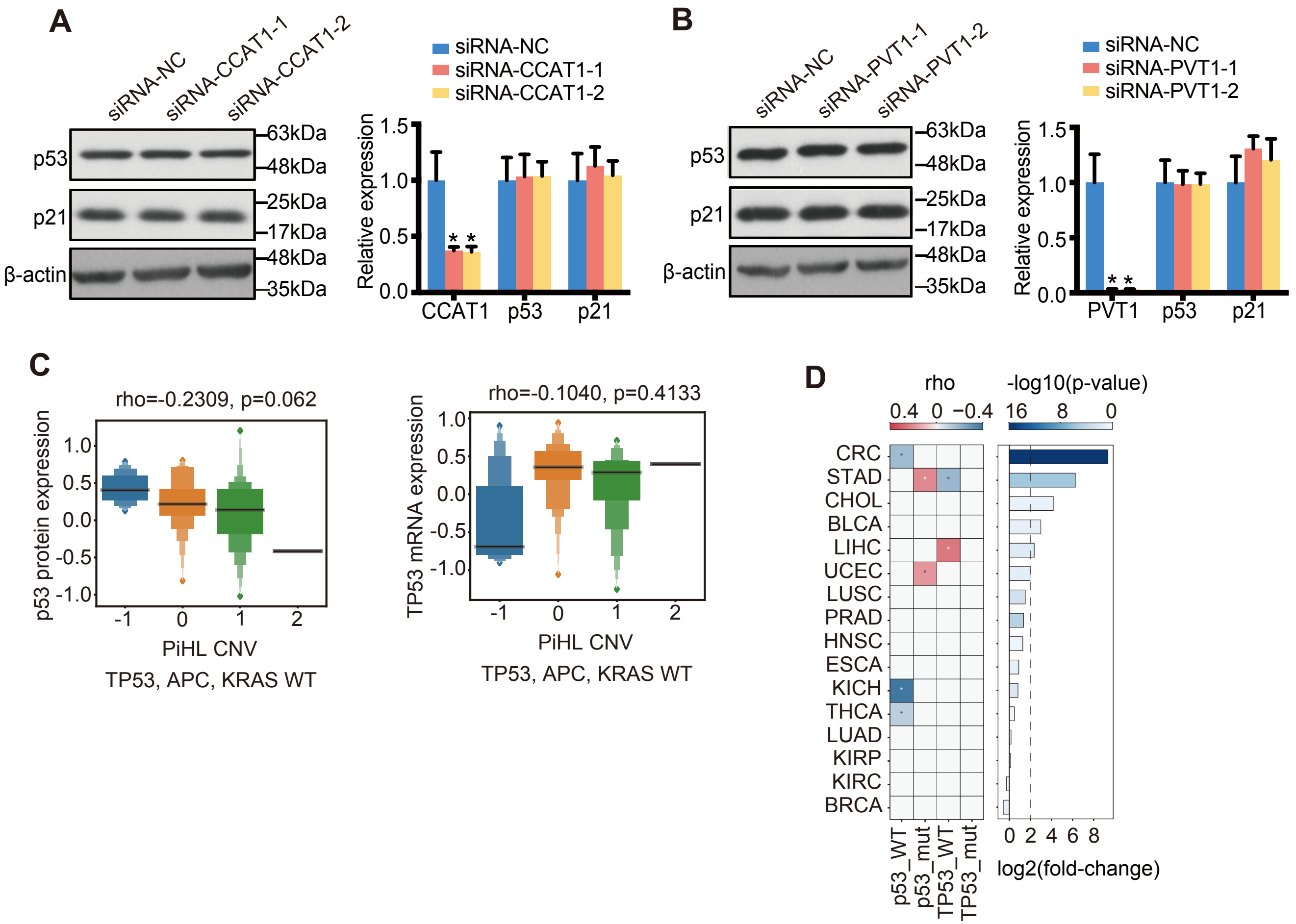

Supplementary Figure 3

A

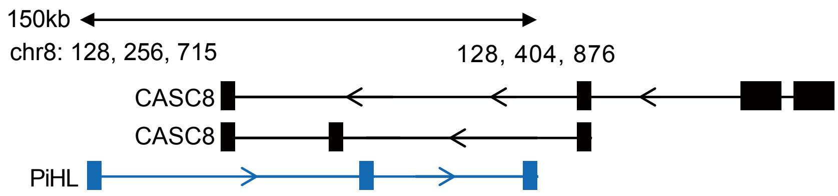

B

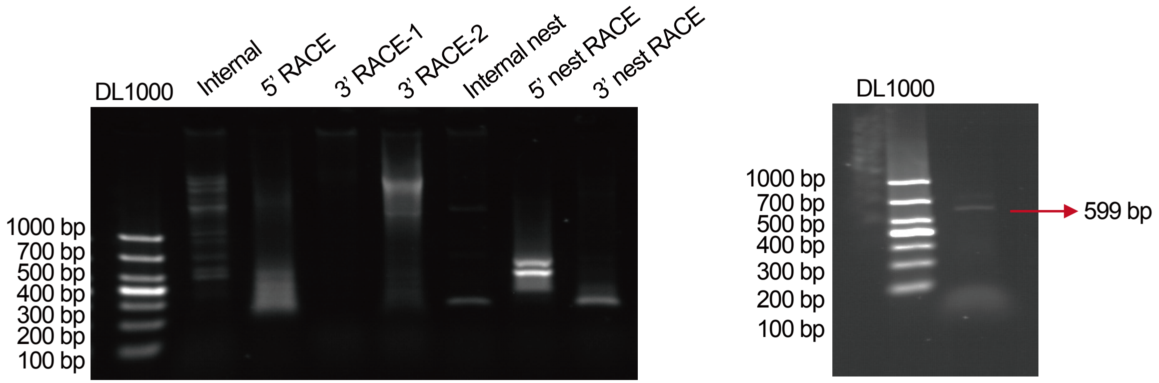

5' GAACAGGTTGTGTGTGCCCCTTGAGGCGTTCATCCAGCACTGTTTCAGAGAAATCCCTATTTCAATCTAT
TCCTATACGTTAGTTATTGAAAAGCAATAGACAATCACAAAAAACAAGTTGACCTTTTTGTGTTCCTTGAGCC
AAAGGGCCCTCATGACTGGGCCTCACACCGAATAACTCGTTACAAAAAGAGCTAGGGTCCCAGACTGCGC
CAAAGCTTCAGGAGACTGCTCCTCGTCTGTGCACAGATGAGTGGCCAACTCTGGAGCCCAGGTTGTTGCT
TCCTAGTCTGGTGGTGAATCCTTCATAGTCTGGAGGTGCTTATTTAGCAAATTCAACCTTAAACCTGAGTGC
ATGGAAACTATTGATGCAGTGTCCAAGGTGGAGAAAGGTCAGAGTGGATCCAGAGGAGCCAAGAGAAGAC
GTCCAGCATGGTGACCTGGGCTCAAGTCAAGGTCCTTATTTCTCTGCTGAACCATCACATTCCTTATACAGA
GTGGGACAGCATTGGCTGAGTTCTACTAGCAAATGCCGGAGATGTGGTTCCTGTTGGCCTTTTAACTGATG
TGATGAAATAAAGTCTTCCATAATTTGACAA **3'**

C

RNA size	ORF size	Ficket Score	Hexamer Score	Coding Probability	Coding Label
599	198	0.8724	-0.123885285535	0.016799578332951	no

D

Label	Strand	Frame	Start	Stop	Length (nt/aa)
ORF1	+	1	433	516	84/27
ORF2	+	2	356	439	84/27
ORF3	+	3	369	566	198/65
ORF4	-	2	589	461	129/42
ORF5	-	3	480	394	87/28
ORF6	-	3	156	73	84/27

Supplementary Figure 4

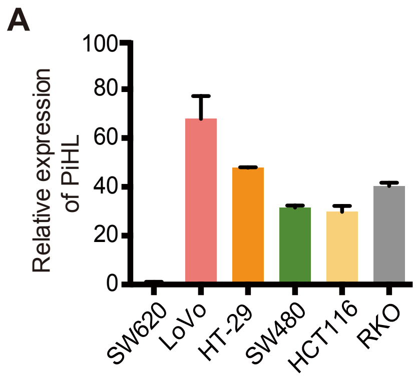

B

Cell lines	PiHL Copy number per cell
SW620	1.59 ± 0.15
LoVo	158.62 ± 14.15
HT-29	86.74 ± 4.48
SW480	31.42 ± 3.2
HCT116	30.26 ± 6.85
RKO	61.12 ± 5.51

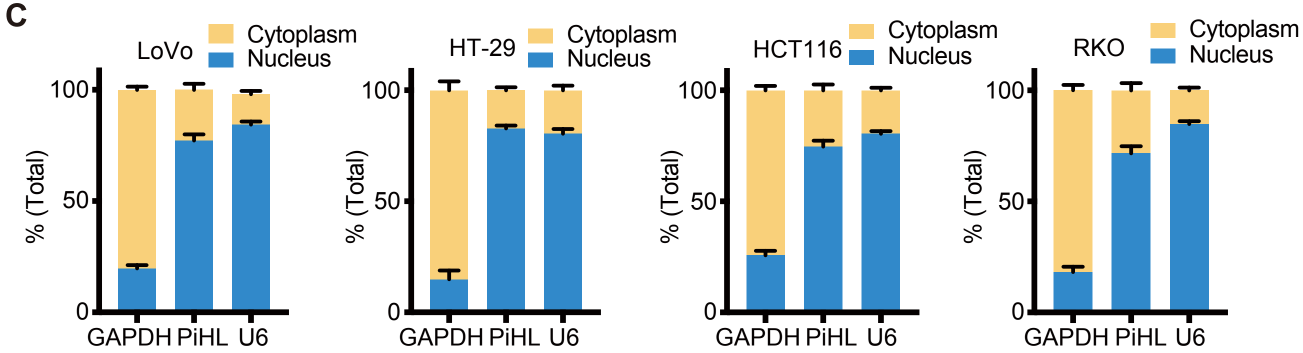

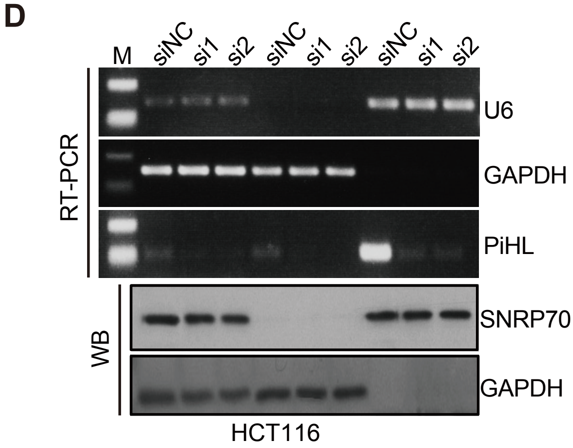

Supplementary Figure 5

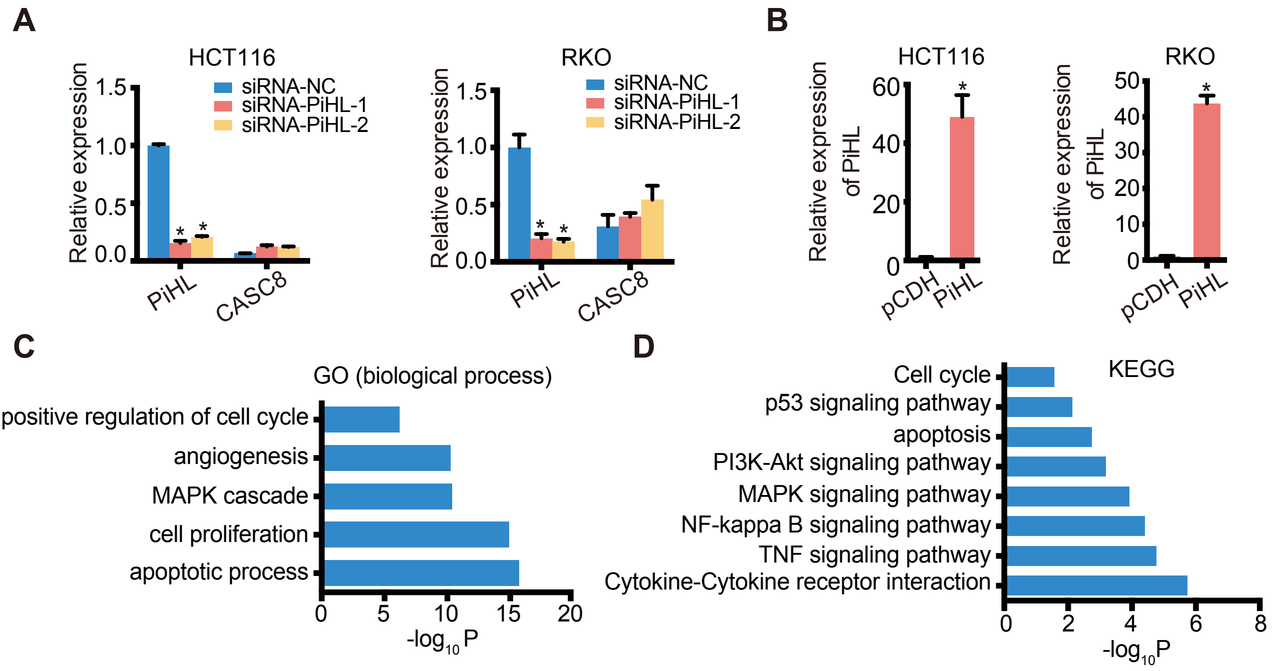

Supplementary Figure 6

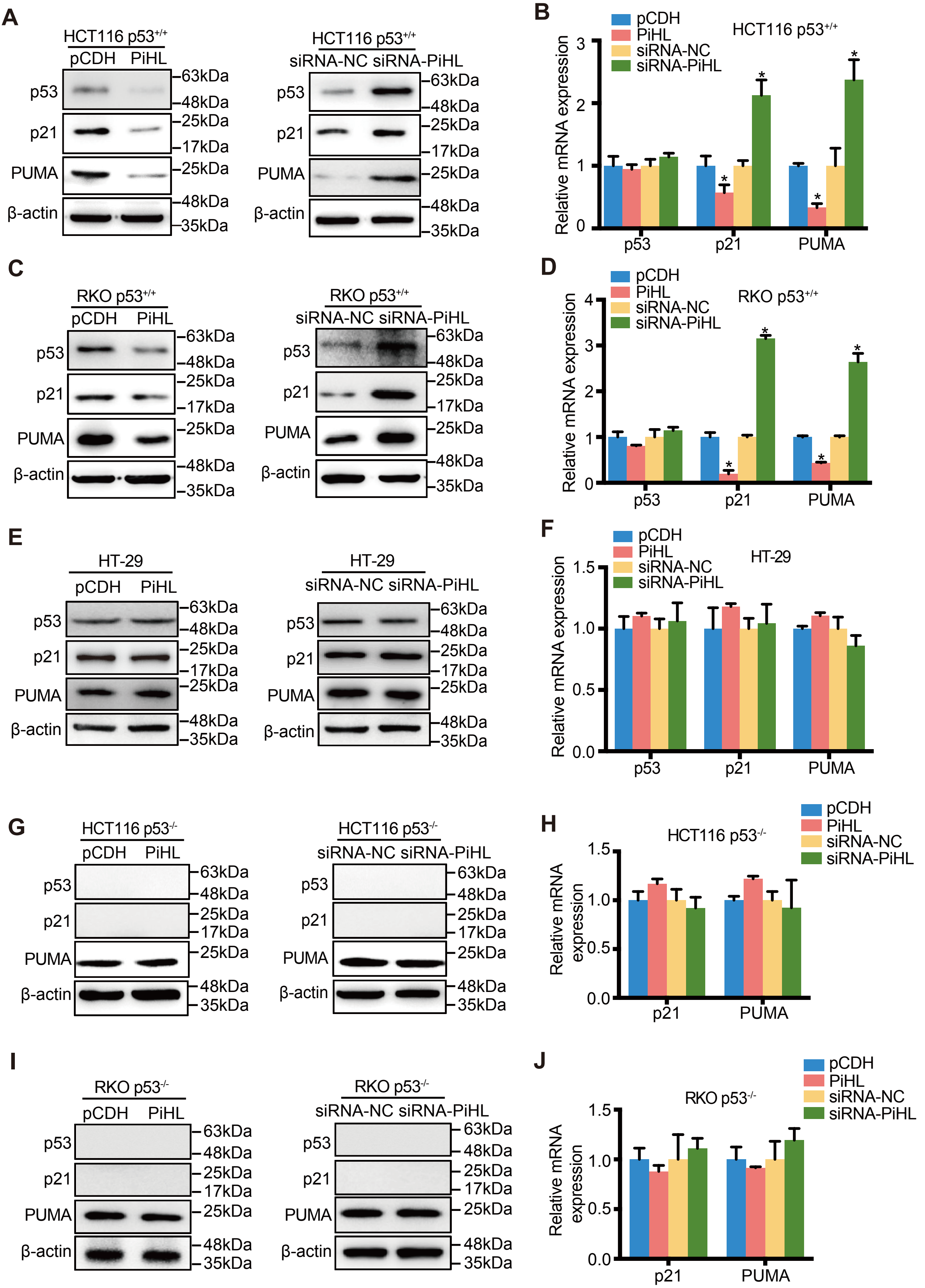

Supplementary Figure 7

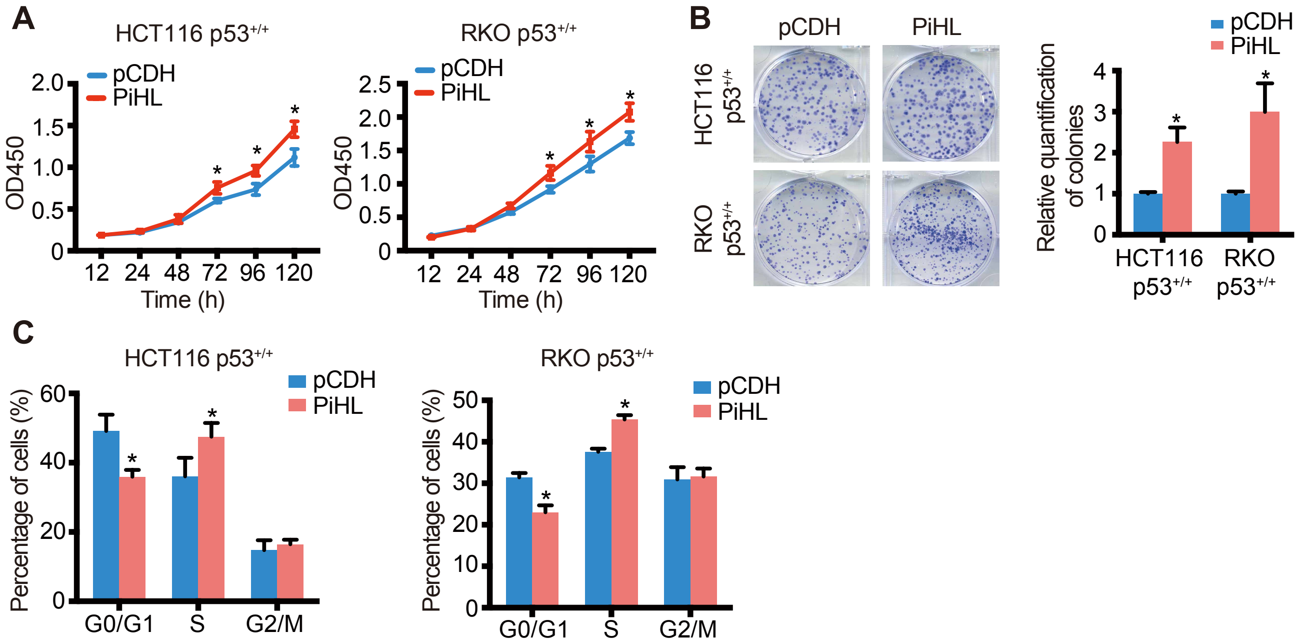

Supplementary Figure 8

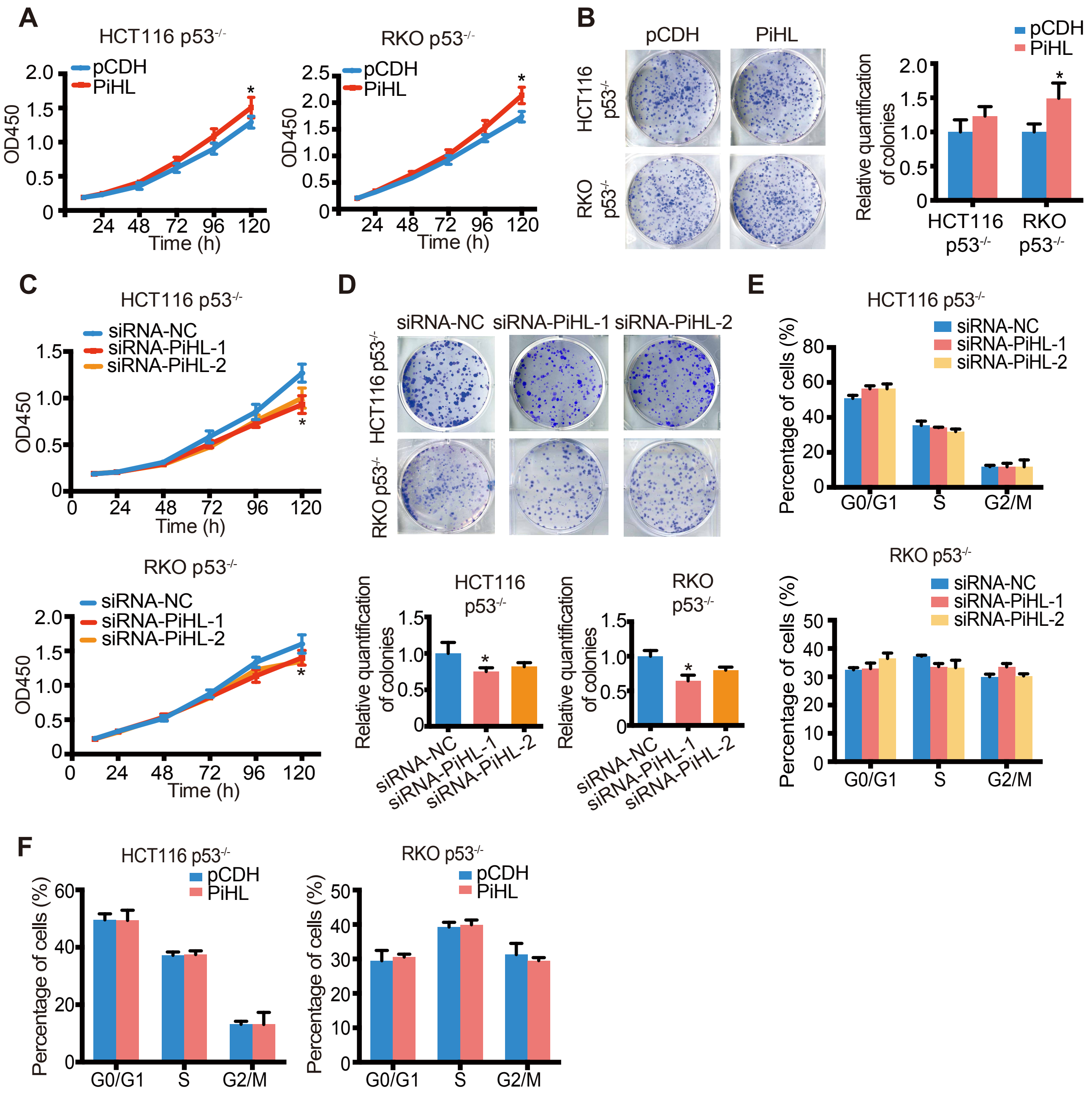

Supplementary Figure 9

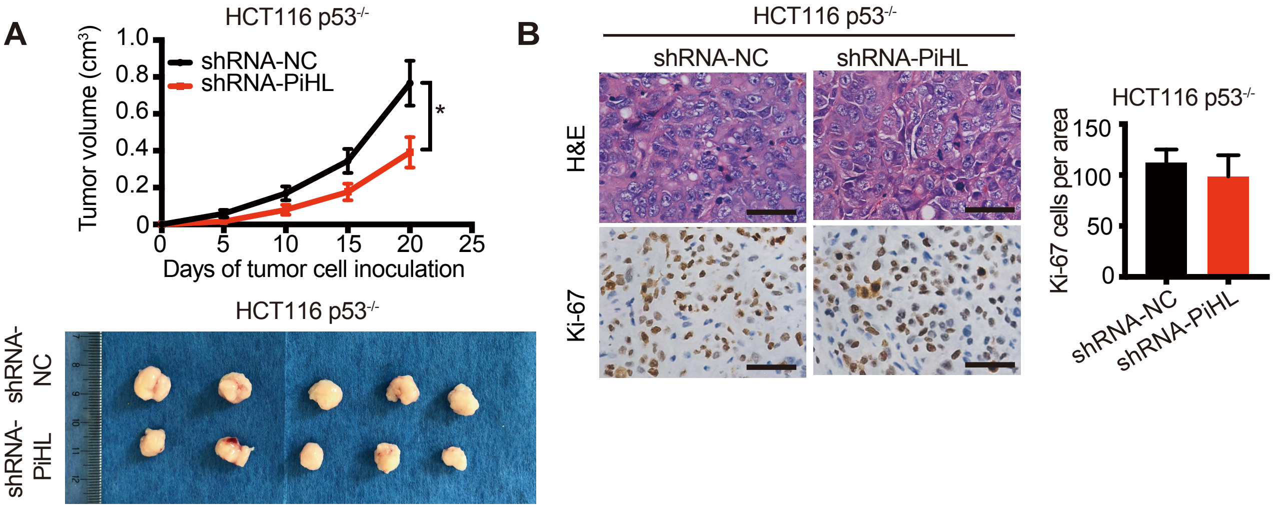

Supplementary Figure 10

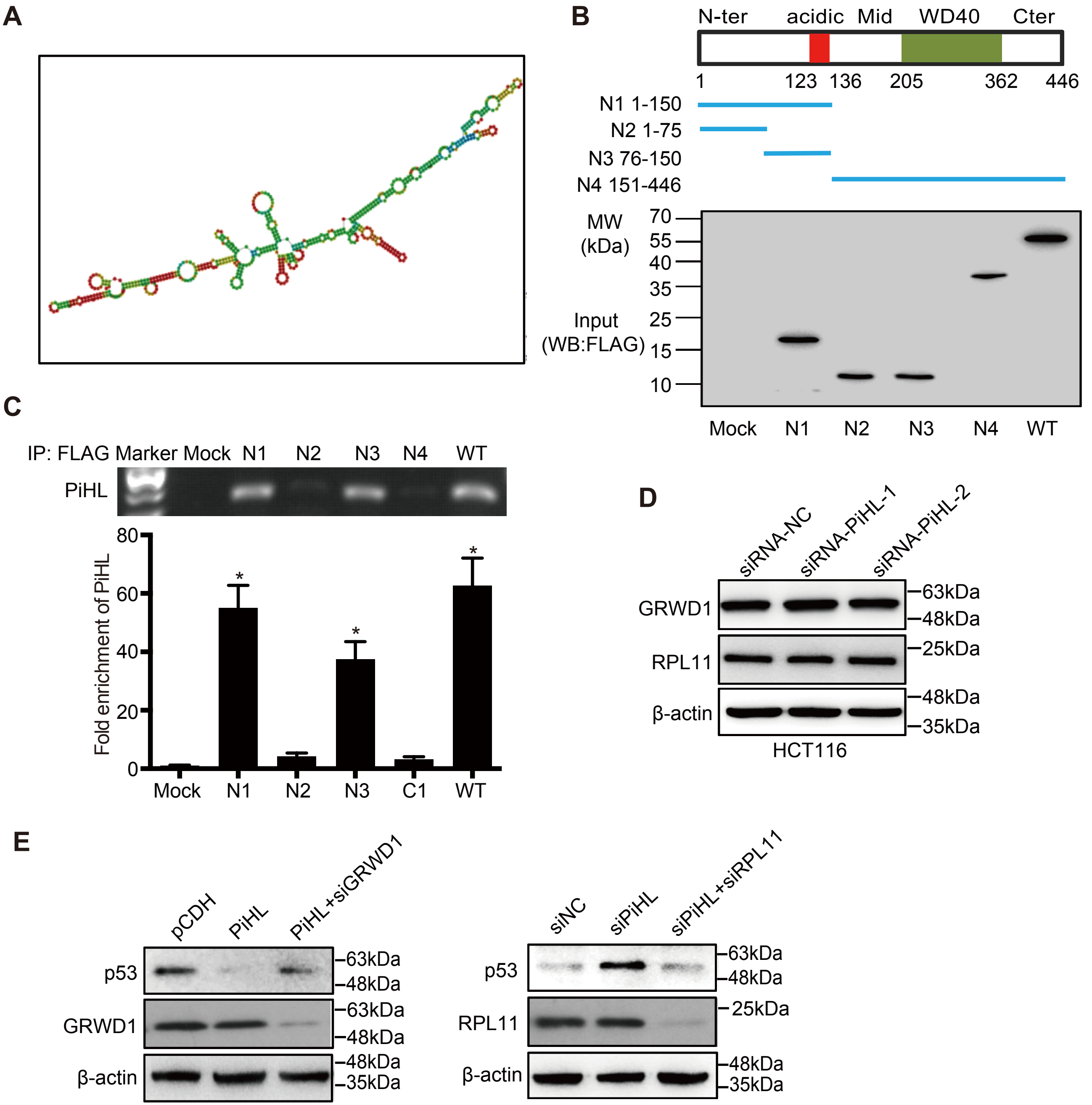

Supplementary Figure 11

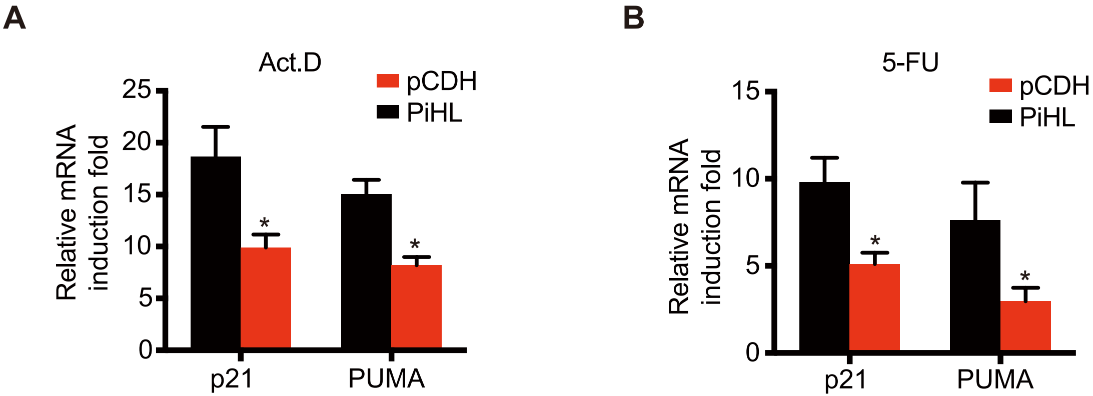

Supplementary Figure 12

A

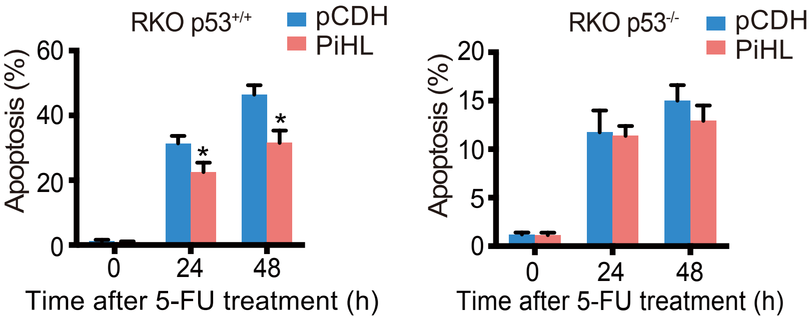

Supplementary Figure 13

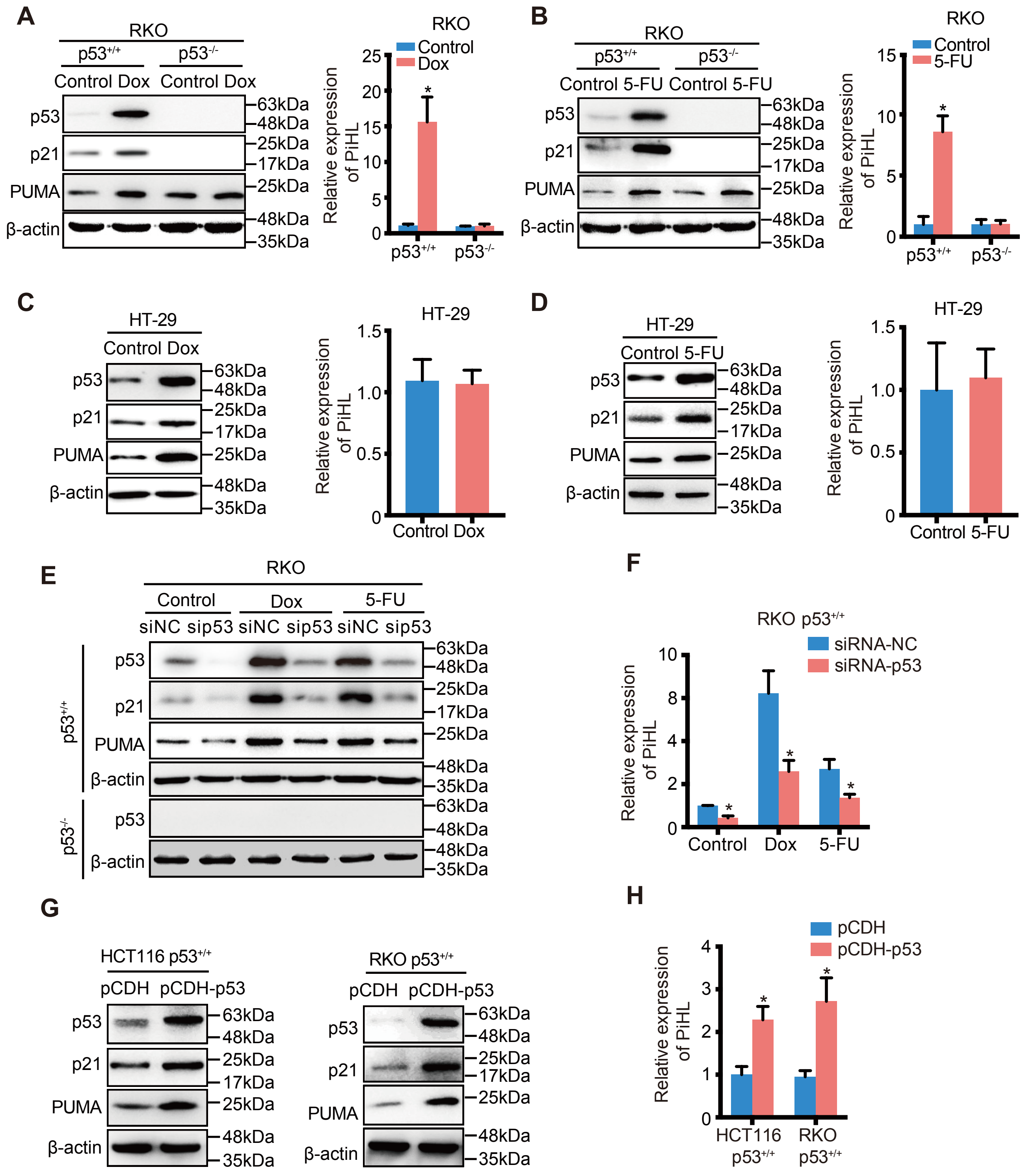

Supplementary Table S1. Primers used in this study.

**Primers used for RACE-PCR**		
Name	sequence	
lncRNA-PiHL-Internal-Forward	AAACTATTGATGCAGTGTCCAAGGTGG	
lncRNA-PiHL-Internal-Reverse	ACATCAGTTAAAAGGCCAACAGGAACC	
lncRNA-PiHL-5′ RACE-GSP	CCTTGACTTGAGCCCAGGTCACCATGC	
lncRNA-PiHL-5′ RACE-NGSP	ACGTCTTCTCTTGGCTCCTCTGGAT	
lncRNA-PiHL-3′ RACE-GSP	GCATGGTGACCTGGGCTCAAGTCAAGG	
lncRNA-PiHL-3′ RACE-NGSP	CTTATTTCTCTGCTGAACCATCACATTCC	
**Primers used for quantitative RT-PCR**		
Name	Forward	Reverse
lncRNA-PiHL	GAGCCAAGAGAAGACGTCCAG	AAAGGCCAACAGGAACCACAT
GAPDH	TCACCACCATGGAGAAGGC	GCTAAGCAGTTGGTGGTGCA
U6	CGCTTCGGCAGCACATATA	TTCACGAATTTGCGTGTCAT
β-actin	AGTTGCGTTACACCCTTTCTTG	GCTGTCACCTTCACCGTTCC
TP53	ACCTATGGAAACTACTTCCTGAAA	CTGGCATTCTGGGAGCTTCA
P21	TCACTGTCTTGTACCCTTGTGC	GGCGTTTGGAGTGGTAGAAA
PUMA	CGGAGACAAGAGGAGCAG	GGAGTCCCATGATGAGATTG
PVT1	GCCCCTTCTATGGGAATCACTA	GGGGCAGAGATGAAATCGTAAT
CCAT1	CCACGTGCACATATTTGAATTG	TGCATTCCCTGCTTAATACTCA
CCAT2	CCGAGGTGATCAGGTGGACTTTC	GTCTTCTGGGCTGATGTTGC
MYC	AATGAAAAGGCCCCCAAGGTAG	GTCGTTTCCGCAACAAGTCCT
**Primers used for different loci of PiHL promoter**	Forward	Reverse
pGL3-lncRNA-PiHL promoter-1-2000	CTAGCTAGCCATCTACATCCTCTGAGTAAAACAGAACCAAA	CCGCTCGAGTGGGACTTGGTGACCATTGTTTGTG
pGL3-lncRNA-PiHL promoter-1-1055	CTAGCTAGCGAGGATTCAAGGATTCCAGTTCTGTTTTACTT	CCGCTCGAGTGGGACTTGGTGACCATTGTTTGTG
pGL3-lncRNA-PiHL promoter-1-615	CTAGCTAGCTCAGACTGGTTCTTCTCGTAAACAAAGG	CCGCTCGAGTGGGACTTGGTGACCATTGTTTGTG
pGL3-lncRNA-PiHL promoter-BR1-mutant	CATGTTTTAGCTTCCCTATGACTTGCTCAC	GCTCAGGCTATCTCTCACATGTCACTAC
pGL3-lncRNA-PiHL promoter-BR2-mutant	GGTGAGCTGGAATTTAGATGGTCATGTTG	CCTTGGTGTAAAATGTCAAGCTGGAGGG
**Primers used for ChIP-qPCR**	Forward	Reverse
lncRNA-PiHL-promoter-ChIP-BR1	GCCATGTCTTGGCTTCCCTAT	GTCAAGCTGGAGGGTACAGA

lncRNA-PiHL-promoter-ChIP-BR2	TCCCTAGGCTTTTTCTGAGGA	GAGTGCTTGGGAGGAGTCTG
**Primers used for lncRNA-PiHL deletion mapping**		
Name	Forward	Reverse
lncRNA-PiHL-1-200	GAACAGGTTGTGTGTGCCCCTT	ACCCTAGCTCTTTTTGTAACGAGTTATTC
lncRNA-PiHL-201-458	CCCAGACTGCGCCAAAGCTTCA	ACCTTGACTTGAGCCCAGGTC
lncRNA-PiHL-459-599	CCTTATTTCTCTGCTGAACCATCAC	TTGTCAAATTATGGAAGACTTTATTTC
lncRNA-PiHL-201-599	CCCAGACTGCGCCAAAGCTTCA	TTGTCAAATTATGGAAGACTTTATTTC
**Primers used for GRWD1-domain deletion mapping**		
Name	Forward	Reverse
pCDH-Flag-GRWD1-1-150	ATGGCGGCGCGCAAGGGT	ACCATAGTGGGGCACCATGGCCA
pCDH-Flag-GRWD1-1-75	ATGGCGGCGCGCAAGGGT	GTGATCCCGGACTATGTCAAAGCTG
pCDH-Flag-GRWD1-76-150	CAGCTTTGACATAGTCCGGGATCAC	ACCATAGTGGGGCACCATGGCCA
pCDH-Flag-GRWD1-151-446	GGCATCAACCGAGTTCGGGTGTCATG	TCAGACGCTGATGGTGCGGAAGATGG

Supplementary Table S2. Antibodies used in this study

Antibody	Company	Catalog #	Species	Dilution
				WB
β-actin	Proteintech	HRP-60008	Mouse	1:10000
GRWD1	Abcam	ab188419	Rabbit	1:1000
RPL11	Abcam	ab79352	Rabbit	1:1000
p53	Cell signalling	48818	Mouse	1:1000
p21	Abcam	ab109520	Rabbit	1:1000
PUMA	Abcam	ab9642	Rabbit	1:1000
cleaved PARP-1	Cell signalling	5625	Rabbit	1:1000
FLAG-tag	Cell signalling	14793	Rabbit	1:1000
HA-tag	Cell signalling	3724	Rabbit	1:1000
His-tag	Cell signalling	12698	Rabbit	1:1000
GST-tag	Cell signalling	2624	Mouse	1:1000
Myc-tag	Cell signalling	2276	Mouse	1:1000
ubiquitin	Cell signalling	3933	Rabbit	1:1000
SNRP70	Abcam	ab51266	Rabbit	1:1000
GAPDH	Cell signalling	EPR16891	Rabbit	1:1000

Supplementary Table S3. siRNA sequences used in this study

Name	Target sequence
siRNA-PVT1-1	CAGCUUCAACCCAUUACGAUU
siRNA-PVT1-2	GCCAUCAUGAUGGUACUUUAA
siRNA-CCAT1-1	AAGCAGGCAGAAAGCCGUAUCUUAA
siRNA-CCAT1-2	GAGAAAGAAGGUGUGUACGUGACUU
siRNA-lncRNA-PiHL-1	CGCCAAAGCUUCAGGAGACU
siRNA-lncRNA-PiHL-2	GAGAAGACGUCCAGCATGGU
siRNA-NC	UCCTAAGGUUAAGUCGCCCUC
siRNA-P53	GACUCCAGUGGUAAUCUAC
siRNA-MDM2	CUAUGAAAGAGGUUCUUUU
siRNA-GRWD1	GGGAUGAGCAGGCCCAAAUGAAG
siRNA-RPL11	AUAUGACCCAAGCAUUGGUAUCU
